# Macrophage Polarization as a Target for Colorectal Cancer Treatment Optimization: A Systematic Review

**DOI:** 10.3390/cancers18132049

**Published:** 2026-06-24

**Authors:** Caden Seraphine, Anne Macleod, Tristan Thornsberry, Shalmali Dharmadhikari, Brayden Martinez, Cara Gable, Abigail Chambers, Vaitheesh Jaganathan, Andrew Littlefield, Susan Galandiuk

**Affiliations:** 1 Price Institute of Surgical Research, Hiram C. Polk Jr. MD Department of Surgery, University of Louisville, Louisville, KY 40202, USA; cjsera01@louisville.edu (C.S.); anne.macleod@louisville.edu (A.M.); twthor02@louisville.edu (T.T.); s0dhar09@louisville.edu (S.D.); brayden.martinez@louisville.edu (B.M.); cara.gable@louisville.edu (C.G.); abigail.chambers@louisville.edu (A.C.); vaitheesh.jaganathan@louisville.edu (V.J.); andrew.littlefield@louisville.edu (A.L.); 2 Division of Colon and Rectal Surgery, Hiram C. Polk Jr. MD Department of Surgery, University of Louisville, Louisville, KY 40202, USA

**Keywords:** tumor-associated macrophage, macrophage polarization, immunotherapy, colorectal cancer, microsatellite instability

## Abstract

**Background:** Colorectal cancer (CRC) remains a leading cause of cancer-related mortality worldwide, with poor survival rates of late-stage disease. While immune checkpoint blockade (ICB) therapy has transformed treatment for mismatch repair-deficient (MMRd)/microsatellite instability-high (MSI-H) tumors, most CRC cases are mismatch repair-proficient (MMRp)/microsatellite-stable (MSS) and derive little to no benefit from current immunotherapy regimens. Tumor-associated macrophages (TAMs) constitute a significant component of the tumor microenvironment (TME) and exhibit a phenotypic gradient between pro-inflammatory (M1-like) and anti-inflammatory, immunosuppressive (M2-like) states. Although their polarization status is increasingly recognized as a key modulator of immunotherapy efficacy in CRC, a comprehensive synthesis of the literature regarding macrophage polarization and its relevance to improving CRC immunotherapy remains lacking. **Methods:** A systematic literature search was conducted across PubMed, EMBASE, and ScienceDirect from inception to December 2025 using terms encompassing macrophages, immunotherapy, immune checkpoint expression, colorectal cancer, and microsatellite stability status. Title, abstract, and full-text screening were performed independently by multiple authors. Sixty-five studies were included following PRISMA guidelines. The protocol was prospectively registered on PROSPERO (ID: CRD420251244320). **Results:** Three key themes were identified: (1) macrophage-mediated mechanisms of resistance to ICB, including M2 polarization driven by the PI3Kγ, STAT3, mTOR, and SIRT-1 axes, immunosuppressive cytokine production (IL-10, TGF-β), and altered immune checkpoint ligand expression; (2) macrophage polarization status and associated biomarkers as prognostic indicators of therapeutic response; (3) emerging macrophage-targeted therapeutic strategies in ongoing clinical trials, including CSF1R inhibitors, CD40 agonists, CD47/SIRPα blockade, and STING agonists. **Conclusions:** TAM polarization is a critical determinant of immunotherapy resistance and patient prognosis in CRC. Macrophage-targeted strategies, particularly M2-to-M1 repolarization approaches used in combination with existing ICB regimens, represent a promising avenue for expanding immunotherapy efficacy beyond MSI-H disease. Further translational research and randomized controlled trials are needed to validate these targets clinically.

## Simple Summary

Colorectal cancer is one of the most common and deadly cancers worldwide, yet most patients do not respond to modern immunotherapy treatments. This seems largely due to the environment surrounding the tumor that actively suppresses the body’s immune response, preventing it from attacking cancer cells effectively. Macrophages, which can exist in either a tumor-promoting or tumor-inhibiting state, are a key player in this environment. This review examines how tumor-promoting macrophages help colorectal cancers evade treatment, their potential use as markers to predict patient outcomes, and emerging therapies designed to alter them to improve colorectal cancer treatment. By consolidating current evidence, this work aims to identify new combination treatment strategies that could extend the benefits of immunotherapy to the large proportion of colorectal cancer patients who currently have no effective immune-based treatment options.

## 1. Introduction

Colorectal cancer (CRC) remains the third most diagnosed cancer in the US in both sexes, with 158,850 new cases and 55,230 deaths from CRC predicted in 2026 [1]. Globally, it is predicted that there will be 3.2 million new cases and 1.6 million deaths by 2040, primarily in high-income countries [2]. While screening and early detection has improved outcomes for early-stage CRC [3], survival for recurrent or distant metastatic disease remains poor, with 5-year survival being only 8–14% [2,3,4]. This high prevalence of disease and poor advanced-stage survival rates highlight the need for optimizing current treatment approaches for CRC.

Immune checkpoint blockade (ICB) therapy (e.g., anti-Programmed Death 1/Programmed Death Ligand-1 [PD-1/PD-L1] immunotherapy), in which antibodies are administered to re-activate specific immune cell interactions, is increasingly being utilized as part of standard regimens for several solid organ malignancies [4,5,6,7,8]. In CRC, the success of immunotherapy has been, for the most part, limited to those with mismatch repair-deficient (MMRd) or Microsatellite-Instability-High (MSI-H) tumors [9]. However, while significant responses have been seen in this subgroup of patients, most CRC tumors are MMR-proficient (MMRp)/Microsatellite-stable (MSS) and have had limited benefit from current immunotherapy regimens. Since MMRd/MSI-H tumors comprise only 10–15% of CRC cases, with even lower prevalence in advanced stages, only a small proportion of advanced CRC patients benefit from immunotherapy. This disparity in response to immunotherapy is largely due to the immune heterogeneity of the tumor microenvironment (TME). Consequently, ongoing research is focused on exploring mechanisms to manipulate immune cell behavior and phenotype within the TME to augment therapeutic efficacy [10].

Tumor-associated macrophages (TAMs) comprise a significant portion of the TME [11] and play a significant role in tumor progression, invasion, and response to therapy. Macrophage plasticity enables them to polarize along a spectrum between pro-inflammatory, classically activated (M1-like), or anti-inflammatory, alternatively activated (M2-like) phenotypes. Their phenotype is dependent on cancer and immune cell stimuli of the TME [12]. The prognostic significance of this in CRC at varying disease stages as well as molecular and genetic subtypes is being investigated [13,14].

The genetic and molecular profile of CRC shapes the TME and TAM behavior, distinguishing “immune hot” MMRd/MSI-H tumors from “immune cold” MSS tumors. Due to high mutation burdens, [15] MMRd/MSI-H CRC tumors exhibit dense infiltration of anti-tumor M1-polarized macrophages [16], a key factor in their superior immunotherapy response compared to MSS tumors [17]. Conversely, resistance to immunotherapy is driven by an immunosuppressive environment involving anti-inflammatory macrophages and regulatory immune cells. While the specific role of TAMs in modulating immune checkpoints remains unclear [18], identifying reliable prognostic markers for immunotherapy response is a critical clinical priority in CRC research [19].

Early trials of immunotherapy in CRC examined metastatic disease, with specific investigation into the PD-1/PD-L1 mechanism of the immune checkpoint blockade. Recent studies have highlighted the importance of macrophages in regulating PD-L1 expression within the TME. While the role of T cells in driving PD-L1 expression is well established, the role of macrophages within this process is less well understood [20]. Additionally, macrophages secrete cytokines known to promote PD-L1 expression in tumor cells [21,22], suggesting that macrophages could be important regulators of immune checkpoint activity in CRC. Targeting TAM signaling pathways might therefore improve the effectiveness of immunotherapy. However, there are still significant gaps in understanding the exact mechanisms by which macrophages influence relevant pathways in CRC.

The objective of this systematic review was to analyze the literature regarding the role of TAMs in the CRC response to immunotherapy, with a focus on mechanisms of resistance to therapy. This included assessing the utility of macrophages as prognostic markers and their potential as therapeutic targets for optimizing immunotherapy response.

## 2. Materials and Methods

### 2.1. Search Strategy and Study Selection

An electronic literature search was conducted using Science Direct, EMBASE and PubMed for relevant articles using the search terms: “macrophages” AND “immunotherapy” OR “immune checkpoint expression” AND “cancer” OR “microsatellite stability” OR “microsatellite instability” from inception to December 2025. Titles and abstracts were screened by inclusion/exclusion criteria by authors independently (CS/TT/SD) and identified for a full text review. Following a full text review, articles were identified for inclusion and data extracted by authors independently (CS/TT/BM). Additional articles were identified from review articles and reference lists of selected studies.

This systematic review was conducted in accordance with the Preferred Reporting Items for Systematic Reviews and Meta-Analyses (PRISMA) guidelines [23] (File S1) and the protocol prospectively registered on PROSPERO (ID: CRD420251244320) [24].

### 2.2. Inclusion Criteria

Inclusion criteria involved original research or review articles on the role of macrophages in immunotherapy response in cancer. The discussion of microsatellite stability status in relation to CRC treatment was also a key factor for inclusion.

Relevance to CRC immunotherapy was considered when weighting collected studies. However, studies conducted in non-CRC solid tumors were included when they provided mechanistic context directly relevant to pathways related to CRC. The cancer and model system for each included system were recorded during data extraction.

### 2.3. Exclusion Criteria

Studies were excluded if they did not primarily focus on solid organ cancers pertaining to TAMs and response to immunotherapy. Studies focused exclusively on chemotherapy or general cell death mechanisms were excluded. Additionally, publication types including editorials, commentaries, protocols, conference abstracts, or letters to the editor were excluded.

### 2.4. Data Extraction

Data from each study included were extracted using a predefined standardized form to record details of the study design, patient/subject population and relevant results, key outcomes, and relevance to cancer treatment. The web-based tool Endnote 21 (Clarivate, London, UK) was used to manage the screening process (https://endnote.com). This included removal of duplicates, title and abstract screening, inclusion and exclusion documentation, full-text access, and reference management. As the heterogeneity of included study designs precluded statistical pooling, no effect measures were specified, and findings were presented as a narrative synthesis.

### 2.5. Risk of Bias and Quality Assessment

Given the heterogeneity of the study designs included in this systematic review, risk of bias and methodological quality were assessed using design-specific instruments. This was applied to primary research studies contributing extractable data to the synthesis. Narrative reviews and background literature cited for context were not subject to formal risk of bias scoring, as the tools used were designed for primary study designs. Two reviewers independently appraised all included studies, with discrepancies resolved through discussion and consensus. The tools applied to each study type are described below. The full risk of bias results can be found in the Supplementary Materials.

The methodological quality of murine studies was evaluated using the SYRCLE risk of bias tool for animal studies [25], developed specifically to assess internal validity in preclinical in vivo research. Each study was appraised across ten domains: (1) randomization of animal allocation; (2) blinding of personnel and study team; (3) blinding of outcome assessors; (4) completeness of outcome data; (5) selective reporting of outcomes; (6) other sources of bias; (7) animal selection bias; (8) standardization of animal handling and care procedures; (9) control of environmental conditions; (10) appropriateness of statistical analysis. Each domain was rated as low, moderate, or high risk of bias.

The methodological quality of the cohort and observational studies was assessed using the Newcastle–Ottawa Scale (NOS) [26]. Studies were assessed on selection of study groups, comparability of cohorts, and adequacy of outcome ascertainment. A score (with a 9-point maximum) was given to each study, where seven to nine points indicated low risk, four to six points indicated moderate risk, and zero to three points indicated high risk of bias.

Given the absence of a universally validated risk of bias instrument for bioinformatics and database analyses, methodological quality and relevance were assessed using a structured four-domain framework adapted for computational and large-scale data studies. This parallels previous works in similar fields, and was adapted with relevance to the current study [27]. Each study was evaluated across the following domains: (1) data source quality, encompassing whether the dataset was publicly validated and of adequate sample size; (2) analytical transparency, including reproducibility of methods and availability of code or raw data; (3) external validation, assessing whether findings were confirmed in an independent cohort; (4) outcome relevance, reflecting the degree to which reported endpoints directly addressed the review question.

The risk of bias of randomized controlled trials was assessed using the Cochrane risk of bias tool [28]. Each trial was appraised across domains including randomization sequence generation, allocation concealment, blinding of participants and personnel, blinding of outcome assessors, completeness of outcome data, selective outcome reporting, and other potential sources of bias. Each domain was rated as low, moderate, or high risk of bias.

### 2.6. Data Synthesis

A narrative synthesis approach was employed due to the substantial heterogeneity in study designs, populations, and outcome measures across included studies. Formal exploration of heterogeneity through sensitivity analysis was not conducted due to the degree of heterogeneity and low number of clinical studies. Several active clinical trials remain unpublished or incomplete at the time of this review. Formal certainty of evidence assessment (e.g., GRADE) was not applied for the same reason.

## 3. Results

**Figure 1:**
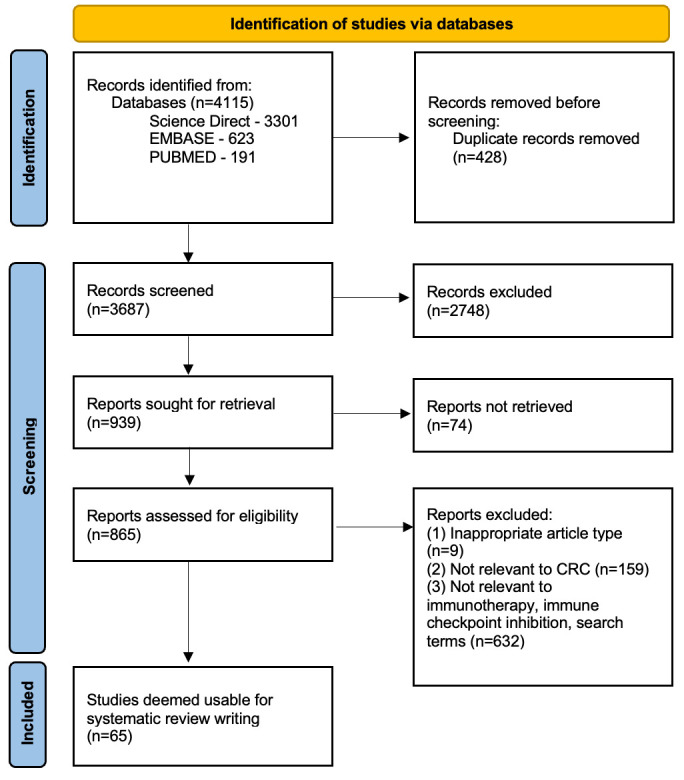
Preferred Reporting Items for Systematic Reviews and Meta-Analyses (PRISMA) flow diagram of selection of included studies [23]. CRC: Colorectal Cancer; Science Direct/EMBASE (Excerpta Medica database)/PubMed databases used for search.

A total of 4115 papers were identified, and following removal of duplicates, 3687 records were screened by abstract for inclusion. Studies retrieved for full text review but not meeting inclusion criteria were excluded primarily due to non-macrophage immune focus, exclusive evaluation of chemotherapy or general cell death mechanisms, or ineligible publication type. From the 865 retrieved for full text review, 65 were included in this analysis (Figure 1). The analysis of 65 results focused on three key themes on the topics of (i) macrophage-mediated mechanisms of resistance to immunotherapy, (ii) the role of macrophages as prognostic markers in colorectal cancer and response to therapy, and (iii) macrophage polarization therapy as an adjunct for colorectal cancer treatment (including current clinical trials).

### 3.1. Mechanisms of Resistance to Therapy

**Figure 2:**
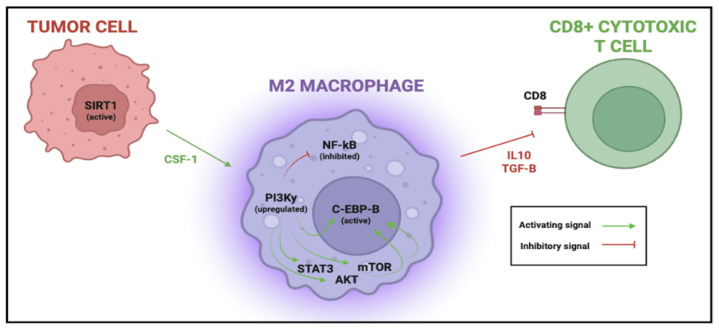
Macrophage-mediated immunosuppression in the colorectal cancer TME. Tumor-derived colony-stimulating factor (CSF-1) promotes the recruitment and polarization of tumor-associated macrophages (TAMs) toward a pro-tumoral M2-like phenotype. This is driven by an intracellular signaling network within TAMs in which PI3Kγ plays a central orchestrating role: by activating AKT and mTOR while simultaneously suppressing NF-κB, PI3Kγ shifts the transcriptional balance away from pro-inflammatory M1 signaling and toward C/EBPβ- and STAT3-driven M2 gene expression. Consequently, M2-like TAMs secrete the anti-inflammatory cytokines IL-10 and TGF-β, which suppress CD8+ cytotoxic T-cell activity and facilitate immune evasion within the TME. Therapeutic inhibition of PI3Kγ has been shown to reverse this signaling bias, reprogramming TAMs toward an anti-tumor M1-like state and restoring immune surveillance (created with Biorender.com).

Within the studies analyzed, 20 different papers were identified studying macrophage driven mechanisms and axes by which resistance to immunotherapy occurs. These included six murine-model experiments, ten patient or clinical sample studies, two preclinical experimental studies, and two computational or multiomics analyses. A summary of these is illustrated in Figure 2.

#### 3.1.1. Polarization Towards Immunosuppressive Macrophage Phenotype

Several intracellular pathways and axes are intricately linked in controlling macrophage function and phenotype, including NF-kB, STAT3, and PI3K-AKT-mTOR. These pathways contribute toward the polarization of macrophages towards an immunosuppressive M2 state, the presence of which are characteristic of immunotherapy-resistant tumors. Inhibition of these pathways can potentially reverse M2 polarization and drive macrophages to their pro-inflammatory phenotype [29,30,31,32].

Macrophage PI3Kγ promotes immune suppression by inhibiting NF-κB (immunostimulant) while activating C/EBPβ (immunosuppressant), thereby creating a pro-tumorigenic environment. Kaneda et al. demonstrated in murine solid tumor models that inhibition of PI3Kγ reversed this mechanism, restoring inflammatory signaling and CD8+ T-cell-mediated anti-tumor immunity. The combination of PI3Kγ inhibition and ICB therapy demonstrated tumor regression and increased survival in a mouse model [33]. Given the expression of PI3Kγ in CRC-associated macrophages, extrapolation of this mechanism to CRC is biologically plausible. Direct CRC-specific validation is needed.

Joshi et al. report a macrophage Syk-PI3Kγ signaling axis that drives immunosuppression in tumors and demonstrated that inhibiting either or both kinases repolarizes macrophages toward a pro-inflammatory, anti-tumor phenotype by activating NF-κB signaling. The authors developed SRX3207, a first-in-class dual Syk/PI3Kγ inhibitor, that promotes CD8+ T-cell activity and anti-tumor immunity [29].

The metabolism regulator, mTOR, has been implicated in M2 macrophage polarization. Inhibition of this regulator has shown to repolarize macrophages from the M2-like phenotype to the M1-like phenotype. mTOR signaling can also promote macrophage-induced angiogenesis via STAT3 [30].

Another key regulator of M2 presentation is the expression of SIRT-1, a class III histone deacetylase. Overexpression of SIRT-1, specifically in CRC tumors, led to an increase in M2 macrophages. Additionally, increased SIRT-1 expression is positively correlated with the activity of immunosuppressive cytokines IL-10 and TGF-β, which are associated with M2 macrophage polarization [31]. Furthermore, M2 and anti-inflammatory markers were also upregulated in cells that were positive for PI3Kγ.

Beyond TAM-intrinsic polarization pathways, tumor-cell intrinsic genetic alternations can also contribute to immunosuppressive and resistant phenotypes. Disturbances in pathways such as Wnt, TGF-β, and TP53 occur within the tumor and contribute towards the limited response of most CRC to immune checkpoint inhibition (ICI). Intrinsic β-catenin dysfunction stemming from the Wnt pathway has shown potential in exhausting T-cells, rendering them incapable of mounting an effective anti-tumor response. Originally described in melanoma [34], this dysfunction has been implicated in CRC immune evasion, but the TAM-specific contribution to CRC remains less well-characterized. Similarly, tumor-cell intrinsic loss of PTEN (phosphatase and tensin homolog) has been associated with decreased CD8+ T-cell infiltration and increased immunosuppression [30]. These findings suggest that PTEN loss contributes to immune resistance by reshaping the TME rather than through a direct effect on macrophage polarization [35].

#### 3.1.2. TAM Immunosuppressive Cytokine/Chemokine Production and Signaling

**Figure 3:**
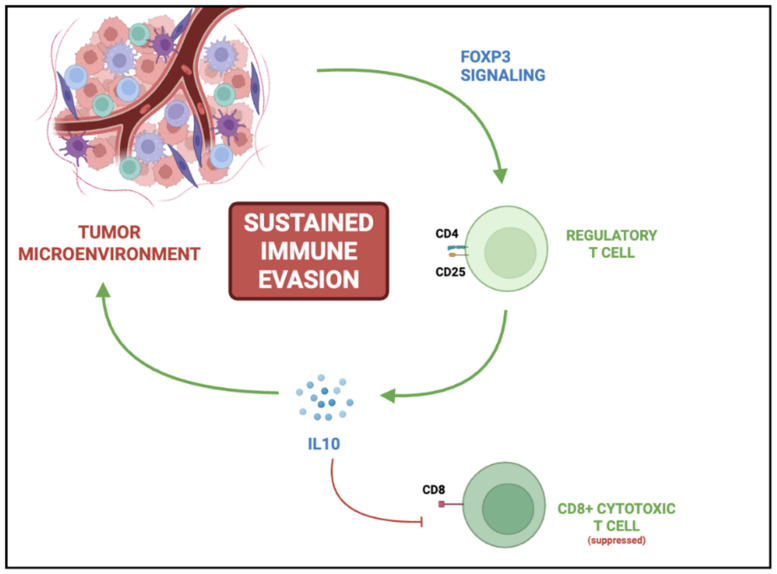
FOXP3-mediated immune evasion feedback loop in CRC TME. FOXP3 signaling drives the development of CD4+ CD25+ regulatory T cells (Tregs), which serve as a key source of immunosuppressive IL-10. This directly suppresses CD8+ cytotoxic T-cell activity via an inhibitory signaling axis, while simultaneously reinforcing the immunosuppressive TME. The resulting feedback loop perpetuates a state of sustained immune evasion, in which Treg-derived IL-10 continuously dampens anti-tumor immunity and supports a TME permissive to tumor progression. These mechanisms do not act in isolation but converge with broader immunosuppressive programs, including M2 macrophage polarization and cytokine-mediated checkpoint dysregulation, to collectively undermine the efficacy of immune checkpoint blockade in CRC (created with Biorender.com).

Immunosuppression and resistance to immunotherapy can be conferred through several cytokine and chemokine signaling pathways. Numerous studies have highlighted the impact of IL-10 and TGF-β production. Fang et al. demonstrated that overexpression of SIRT1 by patient-derived CRC cells significantly inhibited the infiltration of CD8+ T cells and resulted in increased M2 macrophages within the tumor. Additionally, this increase in SIRT-1 expression has been associated with upregulation of immunosuppressive cytokine activity, specifically with respect to IL-10 and TGF-β [31]. An increase in M2 macrophage density is associated with elevated IL-10 production, which in turn suppresses the IL-12 p40 gene expression. This suppression is mediated in part through STAT3 activation downstream of IL-10 signaling, which dampens the transcriptional activity at the IL-12 p40 promoter—a site that requires NF-κB as a cofactor for full induction [36]. This suppressive environment is further reinforced by FOXP3-mediated signaling, which promotes the development of CD4+ CD25+ regulatory T cells (Tregs) that provide an additional source of IL-10, thereby sustaining a feedback loop of immune evasion [37] (Figure 3).

Chemokine-mediated immune cell recruitment further contributes to immunosuppression and TAM recruitment within the TME. In CRC specifically, CCL2 and CCL4, produced primarily by tumor cells and stromal fibroblasts, recruit M2-polarized macrophages to the TME. This reinforces immunosuppressive signaling and enhances resistance to immunotherapy [38]. We hypothesize CCL22, shown to recruit Tregs via IL-1α in pleural effusion models, may operate similarly in CRC via the TGF-β pathway. This requires further confirmation in CRC-specific models [39].

Regarding STAT 3, a recent investigation involving myeloid-derived suppressor cells (MDSCs) has illustrated a connection between tumor-derived exosomes (TDEs) and immunosuppression via this axis. One study demonstrated that TDE-associated Hsp72 triggered STAT3 activation in a TLR2/MyD88-dependent manner via IL-6 production. Targeting this pathway using dimethyl amiloride improved the efficacy of cyclophosphamide in mouse tumor models, with the mechanism further validated in human MDSCs (demonstrated in murine tumor models and validated in human MDSCs ex vivo, though not specifically in CRC) [40].

Furthermore, gene alterations such as higher transposable element (TE) or “jumping-gene” rates have been shown to hinder the efficacy of the immune response. Immune infiltration is fostered by TEs through IFN signaling while also being associated with increased expression of CD274 (the gene encoding PD-L1) and other immune checkpoint genes within the tumor immune microenvironment. Recent evidence suggests that IL-17 signaling exerts context-dependent effects in CRC. In myeloid cells, IL-17RA-mediated signaling promotes macrophage activation, IL-18 production, and CD8+ T-cell-mediated anti-tumor immunity, showing the complex role this pathway plays in modulating responses to immune checkpoint blockade [41].

#### 3.1.3. Immune Checkpoint and Ligand Expression

Resistance to treatment and immune mechanisms can also occur through altered expression of immune checkpoint proteins. A key checkpoint system is the PD-1/PD-L1 immune axis. While PD-L1 expression is a marker for checkpoint blockade efficacy, its presence alone is insufficient to guarantee a clinical response, particularly in immunotherapy treatment-resistant CRC. A group of collective mechanisms establish a biochemical and physical shield that inactivates tumor-infiltrating lymphocytes, directly correlating with the diminished efficacy of ICB in immunotherapy-resistant CRC [17].

PD-L1 expression itself continues to be explored in the context of ICB efficacy. Yavuz et al. used a breast tissue cancer-associated fibroblasts (CAFs) and PBMC model to demonstrate the ability to recruit monocyte into the TME and polarize TAMs to an immunosuppressive, PD-L1 expressing phenotype [42].

Genetic factors also contribute to overall resistance through immune checkpoint expression. High expression of TE has been associated with increased IFN-related signaling and elevated expression of immune checkpoint genes including PD-L1 within the TME. Zhu et al. showcased that CRC patients presenting with overexpression of these TEs had poorer prognosis but could be potential candidates for anti-PD-1/PD-L1 therapy [43].

Inflammatory macrophage PD-L1 expression can be further enhanced by Lipopolysaccharide and IFNγ acting via TLR4 and STAT1. T helper-1 (Th1) cells were also shown to induce PD-L1 expression and could possibly be involved in a positive feedback mechanism where macrophage PD-L1 could stimulate Th1 cells and inhibit T helper-2 cells [44].

Resistance to anti-PD-L1 therapy can be due to a lack of T-cell infiltration or priming. An analysis of the differences in TME by microsatellite instability can be found in Table 1. Peng et al. showed that a loss of PTEN did not affect overall PD-L1 expression but instead promoted the resistance to direct T-cell mediated tumor killing [35]. Llosa et al. examined a cohort of MMRp and MMRd CRC patient samples. They found that IL-17 expression conferred resistance to ICB in both MMRp and MMRd cohorts that were expected to respond positively to such treatment [45].

#### 3.1.4. Metabolic Programming in the TME

Beyond TAM-specific mechanisms, metabolic competition within the broader TME also contribute to immunotherapy resistance. In colon cancer, tumor cells competitively inhibit glutamine uptake by type-1 conventional dendritic cells (cDC1s) through upregulation of SLC38A2, thereby impairing cDC1-mediated T-cell priming and diminishing the efficacy of immune checkpoint blockade therapy [46].

#### 3.1.5. TAM Relevant Cell-to-Cell Crosstalk

The intercellular communication and contact that TAMs participate in within the TME contributes to immunotherapy resistance and phenotypic outcomes. Chief among the relevant key players in the TME are T cells, with interaction contributing to immunosuppression. These interactions have been implicated in effects such as T-cell exhaustion and suppression. For example, immunosuppressive qualities have been shown to be fostered through the interactions with FOLR2+ macrophages and tolerant CD8+ T cells, exhausted CD8+ T cells, exhausted CD4+ cells, and T reg cells [47]. Additionally, signaling factors expressed on TAMs have been seen with recruitment abilities of immunosuppressive T reg cells. For instance, CCL22 is expressed by TAMs to recruit T reg cells to the tumor site, mediating the TGF-β pathway, and further suppressing the immune response [39]. More specifically, CD4+ and CD25+ T reg development is also contributed to by the FOXP3 protein [37].

TAMs also engage in bidirectional crosstalk with stromal cells within the TME, particularly CAFs. CAFs recruit monocytes into the TME and influence their polarization towards immunosuppressive PD-1 + M2-like macrophages characterized by increased expression of CD163 and CD206. This polarization pattern has been demonstrated in a breast cancer CAF model and is believed to operate similarly in the CRC TME [42], where CAF-TAM crosstalk has been implicated in immunotherapy resistance.

### 3.2. Prognostic Markers

Studies examining the role of macrophage phenotype and behavior as prognostic indicators of therapeutic response were included in this analysis. These encompassed four preclinical studies and seven clinical investigations of patient cohort studies, specifically six retrospective observational studies and one prospective cohort study. A summary of the prognostic markers discussed with relevant findings can be found in Table 2.

#### 3.2.1. Macrophage Polarization Status Markers M1/M2 Ratio

The balance of anti-tumor M1 to pro-tumor M2 macrophages serves as an important prognostic indicator of therapeutic response and outcomes in CRC. Several studies have demonstrated that a higher M1:M2 ratio in the TME is associated with improved patient survival [55,59]. Within this balance, according to an observational study, the expression of M2-like immunosuppressive markers such as MRC1 (CD206) and CD163 indicates a poor prognosis [48].

The transcription factor MAF, a regulator of M2 differentiation, has also been identified as a prognostic indicator. A prospective cohort study reported lower intraepithelial M1:M2 density ratio as indicated by MAF and MRC1 expression correlated with advanced CRC disease stage [60]. Furthermore, a preclinical mechanistic study identified the downregulation of the M2-like marker SPP1 (Osteopontin) as a sign of successful therapeutic repolarization [49]. Although this was demonstrated in a gastric cancer model, this may be able to be extrapolated to CRC.

The broader immune cell composition of the TME, including M0, M1, and M2 macrophages, along with naïve B cells and T-helper cells, constituted risk factors used to predict prognosis in a retrospective observational study [61]. This shift in cellular proportions underscores the functional plasticity of the immune landscape, where the recruitment of suppressive M2-like populations directly impairs the anti-tumor efficacy of resident T-cell and B-cell populations. By integrating these immune signatures into clinical models, the phenotypic state of these markers serves as an indicator of therapeutic resistance and offers a more granular prediction of patient outcomes than traditional histological staging. An example of this is the ‘immunoscore’, a measure of CD3+/CD8+ T-cell infiltration at the tumor core and invasive margin, which is used as a prognostic indicator for risk of recurrence, disease-free survival, and overall survival in stage I–III colon cancer [56]. While not being a measure of macrophage infiltration, studies suggest that combining the immunoscore with macrophage phenotyping—particularly M1/M2 polarization markers (CD80/CD163 ratio) and spatial distribution—could improve risk stratification and treatment selection [11,62].

The expression of molecules that drive M2 polarization also function as prognostic indicators and therapeutic targets. SIRT1, a class III histone deacetylase, has been identified as a marker of poor prognosis in several cancers including prostate, breast, and CRC. SIRT1 is thought to act as tumor-promotor via inhibiting apoptosis and promoting metastasis, andhas been shown to promote TAM recruitment into the TME likely via the CXCR4/CXCL12 pathway [31,63].

Post-transcriptional regulation via microRNAs may further influence macrophage polarization. miRNA-146b, miRNA-155, and miRNA-22 have been associated with modulation of TGF-B signaling and may promote M1 polarization, with potential implications for enhancing responsiveness to checkpoint blockade immunotherapy [52].

Macrophage surface receptor expression also represents prognostic vulnerability. Colony-stimulating factor 1 receptor (CSF1R) is a type III tyrosine kinase receptor found on the surface of myeloid cells. High overall or macrophage CSF1R expression in CRC is predictive of poor survival in CRC. Lv et al. demonstrated inhibition of CSF1R using a specific CSF1R inhibitor PXB17, which effectively repolarized M2-like TAMs towards an M1-phenotype via blocked activation of PI3K/AKT/mTORC1 signaling in a preclinical study [50]. Their in vivo work suggested that PXB17 repolarized macrophages were able to induce CD8+ T-cell infiltration and improve the immunosuppressive microenvironment, and subsequently PXB17 enhanced therapeutic activity of anti-PD1 antibodies in a MSS (CT26) model and prevented tumor regrowth in a MSI (MC-38) murine model [50].

Similarly, Epidermal Growth Factor Receptor (EGFR) is of growing interest as a target. It has been found in cell-line and mouse models that EGFR, which is highly expressed in TAMs, unfavorably promotes polarization towards the M2 phenotype. Inhibition of such signaling pathways can direct repolarization towards M1 [51]. Beyond this, it has been seen that using Cetuximab (a monoclonal antibody that binds to EGFR) resulted in macrophage repolarization towards an anti-tumor phenotype, further supporting its prognostic and therapeutic relevance [64].

#### 3.2.2. Soluble Biomarkers and Non-Macrophage Immunoprognostic Factors

The landscape of immunotherapy response is increasingly defined by systemic and cellular biomarkers. Mirroring patterns seen in other gastrointestinal malignancies, baseline levels of IL-8, TIE2 (tyrosine kinase with immunoglobulin-like and EGF-like domains 2, [an endothelial cell marker]), and HGF (hepatocytic growth factor) in esophageal cancer are significantly associated with patient survival and serve as effective predictors of immunotherapy response [53]. Within the CRC microenvironment specifically, the infiltration of anti-tumor CD8+ T cells—particularly those expressing CXCR3—is linked to a favorable prognosis and a high M1:M2 ratio [55]. Although its full prognostic role in CRC is still being elucidated, the CD47/SIRPa axis represents a critical signal for cancer cell evasion of macrophage [57]. Additionally, CD74 has also been reported as a potential predictor of response to cadonilimab (a PD-1/CTLA-4 bispecific antibody), supported by both in vitro co-culture systems and murine models [58].

Notably, tumor-derived cytokines can also shape prognostic outcomes. In CRC, tumor cell-derived GM-CSF promotes M1 macrophage polarization characterized by CD16 expression, which exerts cytostatic effects on tumor cells. High GM-CSF production by tumor cells is an independent favorable prognostic factor, particularly in mismatch repair-proficient tumors with low CD8+ T-cell infiltration [54].

Together, these findings demonstrate that macrophage polarization status, its driving factors, and associated immune biomarkers provide prognostic significance that may better stratify macrophage-targeted and immunotherapeutic strategies in CRC.

### 3.3. Macrophage Polarization Therapy—Ongoing Trials

Ongoing or completed randomized control trials (RCTs) and preclinical studies investigating macrophage-targeted and macrophage-modulating therapies in CRC were identified from the reviewed literature and via ClinicalTrials.gov registry (https://clinicaltrials.gov) using the keywords above. The approaches of these trials were categorized into (i) immune receptor checkpoints and co-stimulatory agonists, (ii) receptor and kinase inhibitors, and (iii) cytokines and pattern recognition receptor agonists broad immunomodulatory and metabolic approaches discussed below.

Collectively, these approaches aim to either deplete immunosuppressive TAMs, repolarize anti-inflammatory-like macrophages towards a pro-inflammatory phenotype or enhance macrophage-mediated anti-tumor immunity in combination with ICB therapy. An overview of targets used in current therapeutic trials is found in Table 3 and a detailed summary of trial status for therapeutic targets is found in Supplemental Table S1.

#### 3.3.1. Immune Receptor Checkpoints and Co-Stimulatory Agonists

A major focus of macrophage polarization strategies involves targeting receptors that control macrophage activation and T-cell priming.

A growing approach has been the focus on CD40. Agonistic anti-CD40 antibodies target this key co-stimulatory receptor on antigen-presenting cells, promoting M1 polarization and enhancing cytotoxic T-cell activity. Alongside its effect on other cells of the TME, CD40 agonism has been shown to convert TAMs into an activated phenotype with anti-tumor properties that can promote tumor regression independent of T cells [65]. This makes CD40 agonism particularly relevant to combination therapy. Several recent CD40 agonists have been developed and are undergoing clinical trials in combination with anti-VEGF therapy for metastatic colorectal cancer [66], anti PD-1 inhibitors, and short-course radiotherapy for rectal cancer [65].

CD137 is another co-stimulatory receptor on activated immune cells that is absent from resting cells. CD137 is a favorable biomarker for immune activation, with agonistic CD137-antibody therapy proposed to increase activation and proliferation of CD8+ and CD4+ T cells, upregulate IFNγ production, increase macrophage infiltration, and inhibit the proliferation of myeloid-derived suppressor cells and regulator T cells. Numerous in vitro studies and clinical trials have explored its role as mono- or combination therapy in solid organ cancers, with promising results in mouse models [67]. CD137 agonism was found to enhance anti-PD-1 induced activation of CD8+ T cells in a neoadjuvant pancreatic cancer trial [68]. Its role in combination therapy for CRC has been explored as part of larger solid organ trials. It has demonstrated both safety and tolerability, with no definite evidence of benefit in CRC outcomes; however, further trials are ongoing.

The CD47/SIRPa axis is an innate immune checkpoint exploited by cancer cells to promote immune evasion and tumor progression. Expression of CD47 on cancer cells inhibits macrophage-mediated phagocytosis by binding to SIRPa on macrophages, making the blockade of this pathway a target for novel therapies. Therapies targeting the CD47/SIRPa axis are designed to restore the phagocytic function of macrophages. Anti-CD47 or anti-SIRPa antibodies block the cancer cell immune evasion signal, promote macrophage-mediated phagocytosis, and allow tumor antigen presentation to T cells, leading to secondary activation of CD8+ T cells [69,70]. This all comes in the context of CD47 as particularly expressed in advanced disease, making it a target of combination therapy. In one particular focus, combination of the anti-CD47 antibody magrolimab with the anti-EGFR antibody cetuximab in a Phase1b/2 clinical trial was well tolerated in patients with pre-treated KRAS wild-type CRC, with no response observed in patients with KRAS mutant CRC [71].

Preclinical work continues to leverage intrinsic mechanisms of macrophages to repolarize towards the desired phenotype. Here, stimulator of interferon gene (STING) has become a leading source of investigation, functioning as a critical cytosolic DNA sensor and initiator of a type 1 interferon response to activate innate immunity. In a murine colon cancer model, it was demonstrated that intravenous administration of GB2, a novel STING agonist prodrug targeting triggering receptor expressed on myeloid cells 2 (TREM2), induced tumor regression. When combined with anti PD-1 immunotherapy, a synergistic effect on tumor inhibition and prolonged mouse survival was observed [72], making this increasingly relevant to the improvement of current ICB therapy.

Immune checkpoint inhibition has been shown to be enhanced through TAM repolarization via antibody targeting. Anti-macrophage receptor with collagenous structure (MARCO) monoclonal antibody treatment was developed to elucidate anti-tumor activities. Anti-MARCO treatment enhanced the anti-tumoral effects of anti-CTLA4 antibody therapy in colon carcinomas [73]. With CTLA-4 a key immune checkpoint on T cells that downregulates early stages of the immune response, this dual blockade is a potent strategy for both reprogramming the TME and restoring T-cell effector function.

#### 3.3.2. Receptor and Kinase Inhibitors

Inhibition of specific receptors and kinases has been an alternative approach to both depletion and repolarization of M2 TAMs. CSF-1 secreted by tumor cells bind to CSF1R on macrophages, triggering the intracellular signaling cascade that enables macrophage survival and the polarization towards an M2 phenotype [50]. As discussed, CSF1R inhibitors block this pathway, which is essential for TAM survival and M2 polarization, leading to TAM depletion or repolarization. Other targets include TREM2 Inhibitors and Clever 1 Inhibitors, which block specific receptors implicated in maintaining the immunosuppressive and pro-angiogenic functions of M2 macrophages [67,74,75,76].

Single-cell analysis is being employed to explore receptor interactions in this pathway. One study found that anti-CSF1R treatment demonstrated selective reduction of inflammatory macrophages, while failing to eliminate macrophage subsets associated with tumor growth and angiogenesis in both murine and human models. The same analysis found that CD40 agonism induced dendritic cells to upregulate T-cell infiltration [77].

Again, translational research remains a focus of the analysis of these mechanisms. In this context, a study was conducted examining the effects of cetuximab on the TME of CRC. Cetuximab competitively binds the EGFR-like receptors on cancer cell surfaces, blocking the signaling cascade. Alterations in macrophage polarization were also observed with cetuximab treatment. With treatment, decreases in M2 TAM markers such as Arg1, Mrc1, and IL-10 were found along with observed increases in M1 TAM markers such as iNOS, IL-12, and TNF⍺ [60,78].

#### 3.3.3. Cytokines and Pattern Recognition Receptor Agonists

Other approaches rely on improving current modalities, introducing or activating signals that inherently drive a strong pro-inflammatory M1 response. IFNγ serves as the primary classical activator of the M1 phenotype via signaling through the JAK-STAT1 pathway, while IL-12 acts as a critical bridge to adaptive immunity by promoting Th1 differentiation and reinforcing a positive feedback loop of IFNγ production that enhances anti-tumor cytotoxic response. The direct use of IFNγ/IL12 leverages their power as M1-polarizing cytokines [58]. Similarly, TLR agonists (e.g., targeting TLR 3 and TLR7), which activate this crucial part of the innate immune system’s mechanism for sensing danger, trigger a potent M1-skewing response in macrophages [69,79].

#### 3.3.4. Broad Immunomodulatory and Metabolic Approaches

Several current strategies approach with a systemic impact on the TME and macrophage metabolism. Broad immunosuppressive pathways are currently being targeted, such as the inhibition of TGF-b. Meanwhile, immunotherapy staples like anti-PD-1/PD-L1 checkpoints indirectly affect macrophages by reversing T-cell exhaustion, which can facilitate M1 repolarization [52]. Metabolic inhibitors of lactate pathways aim to target the unique metabolic dependence of M2 cells, while macrophage cell therapy represents a highly innovative approach involving the direct infusion of engineered or ex vivo re-educated macrophages. Complement inhibitors and vitamin E are also being explored for their general immunomodulatory effects on the TME [80]. Yuan et al. reported an improved survival in patients taking vitamin E supplement whilst on immunotherapy and demonstrated an increased anti-tumor efficacy of immunotherapy with vitamin E supplementation in a mouse model. Vitamin E uptake of dendritic cells (DCs) restored their anti-tumor function by inhibiting SHP1, enhancing tumor-antigen cross presentation [81]. While each of these may differ in their focus, the consistent theme of alteration to inflammatory drivers remains consistent in metabolic approaches.

Targeting glucose metabolism enzymes has emerged as a potential strategy to reshape the TME. HKB99, an inhibitor of phosphoglycerate mutase 1, was shown in a preclinical cell and murine mode to increase cytotoxic CD8+ T-cell activity in the TME while also decreasing the concentration of M2-like macrophages. Furthermore, HKB99 further enhanced the tumoricidal effect of immune checkpoint-blocking treatments like anti-PD-1 antibodies on colon cancer models [81,82].

Current clinical efforts focus on enhancing the therapeutic index of immunotherapy (relative safety) through localized activation and high-precision delivery systems. Recent trials emphasize Fc-optimized CD40 agonists and bispecific 4-1BB antibodies designed to mitigate systemic toxicities, while oncolytic viruses and mRNA-based cytokine delivery are increasingly being utilized to convert immunologically “cold” MSS tumors into immunologically “hot” environments. Furthermore, the development of prodrugs targeting TREM2-expressing TAMs and the integration of multi-kinase inhibitors demonstrate a shift toward targeted macrophage repolarization as a primary strategy to overcome the immunosuppressive barriers of the CRC TME.

#### 3.3.5. Alternative Trial Outcomes

Despite promising preclinical models, across the trials reviewed, several therapeutic strategies either failed to deliver meaningful clinical benefits, raised safety concerns, or both. Some of these trials have been highlighted below and are discussed further in Supplementary Table S1.

Despite successes in TGF-β-targeted therapy models, there have also been several cases of limited clinical efficacy. The trial of combination drug Anti-PD-L1/TGFβ Fusion Protein M7824 (NCT03436563) showed limited efficacy and potential adverse effects in metastatic CRC and other advanced MSI-H solid tumors [83]. Several TGF-β-targeting clinical trials have been completed with results pending, evaluating its use alone or in combination with either anti-PD1, anti-PDL1, CD39 or recombinant IL-2 in the advanced or metastatic setting (Supplementary Table S1). This confirms the importance researchers have recognized in targeting TGF-β in clinical trials, but the right combination therapy in the clinical setting has not been identified.

The addition of delta-tocotrienol (vitamin E) to FOLFOXIRI chemotherapy (NCT02705300) failed to extend time to hospitalization or death in metastatic CRC and offered no meaningful reduction in peripheral neuropathy or grade 3–4 toxicities. This offered little benefit beyond standard treatment [84].

The combination of MEK inhibition and anti-PD-L1 (NCT03428126) in MSS CRC has shown limited efficacy [85]. With an overall response rate of just 3.4%, a median progression-free survival of 3.2 months, and no meaningful shift in T-cell infiltration, the combination failed to meet its efficacy benchmarks.

Among examined metabolic inhibitors, the results were consistently limited. The arginase inhibitor INCB001158 (NCT02903914) managed to raise plasma arginine levels but produced only limited anti-tumor activity [86]. CCR2 and CCR5 are known mediators of macrophage migration and TAM infiltration into the TME. In a trial combining CCR2/5 blockade with chemotherapy versus immunotherapy in MSS CRC or pancreatic cancer patients, BMS-813160, a dual CCR2/5 antagonist (NCT03184870), lacked efficacy specifically in MSS CRC [87]. Maraviroc combined with pembrolizumab (NCT03274804) achieved a 5.3% objective response rate, and while there was some evidence of TME modulation, translating that into clinical benefit has been less clear [88].

Perhaps the most consistent treatment failure in clinical trials has come from CSF1R inhibitors, including BLZ945 (NCT02829723), PLX3397 (NCT02452424), AMG 820 (NCT02713529), and ARRY-382 (NCT02880371) [89,90,91,92]. These all exhibited measurable macrophage-targeted activity, but there was less evidence of meaningful clinical outcomes when paired with checkpoint inhibitors. This ultimately resulted in multiple trial terminations.

Lastly, the IL-15 receptor agonist nanrilkefusp-⍺ (NCT05619172) was discontinued after interim data showed no effect, either as a monotherapy or in combination with cetuximab, in RAS wild-type CRC. A broader multi-agent immunotherapy combination trial in MSS metastatic colorectal cancer (NCT03555149) was shut down due to limited efficacy, compounded by recruitment difficulties and resource constraints [93].

### 3.4. Risk of Bias Assessment

Of the 19 mouse model studies evaluated, the majority were rated as low or moderate risk across most domains. Five studies received high risk ratings from both reviewers [29,30,41,44,55]. Areas of methodological concern were acknowledged during qualitative synthesis (Supplemental Table S2a).

Of the 19 included cohort and observational studies, the majority were rated as low or moderate risk by both reviewers using the Newcastle–Ottawa scale. Complete agreement was reached for 16 of 19 studies; 3 studies showed minor inter-reviewer discrepancy of one risk category, which were resolved by consensus. One study [70] was independently rated as high risk by both reviewers, indicating notable methodological limitations acknowledged during qualitative synthesis (Supplemental Table S2b).

For bioinformatics and database analyses, methodological quality and relevance were assessed using a structured four-domain framework adapted for computational and large-scale data studies. In contrast to the risk of bias assessments applied to other study designs, ratings were assigned as high, low, or unclear to reflect the level of methodological quality and relevance rather than risk of bias per se. Both reviewers independently rated all six included studies [32,47,52,60,61,62] as high across all domains, with no discrepancies requiring discussion (Supplemental Table S2c).

All included randomized controlled trials [64,71,83,84,85,86,87,88,93] were independently rated as low risk of bias by both reviewers across all assessed domains, representing the highest level of internal validity among the study designs included in this review (Supplemental Table S2d).

## 4. Discussion

This review investigated the multifaceted role of macrophages in the TME of CRC, modulating immunotherapy efficacy via several key mechanistic pathways, and combination approaches. We also examined therapeutic strategies targeting macrophage polarization, specifically M2-to-M1 repolarization and direct M2 macrophage inhibition.

Examining immunotherapy resistance, we identified several mechanisms by which key components in the TME confer resistance to immune checkpoint blockade therapy for CRC. While the breadth of investigations with varying targets highlights that resistance is multifaceted, it is evident that this resistance is associated with an immunosuppressive environment characterized by dense infiltration of anti-inflammatory macrophages, Tregs, and myeloid-derived suppressor cells. Identified cytokines, including TGFβ and IL-10, seemingly blunt the efficacy of checkpoint inhibition via prevention of T-cell infiltration and function. Such findings suggest that T-cell-focused approaches may be insufficient without adjunct cytokine modulation to alter the composition of tumor infiltrates.

Several molecular pathways directly modulate macrophage function in the TME. PI3Kγ, which inhibitits the immunostimulant NF-kB and activates the immunosuppressant C/EBP-B, is a primary promoter of immune suppression and serves as a potential therapeutic target. This reinforces the argument that optimizing therapeutic efficacy may involve inhibiting these pathways to reverse M2 polarization as opposed to depleting macrophages entirely.

Although the identification of reliable prognostic markers remains an unmet need, the data synthesized supports the value of macrophages in this space and illustrates a connection between both their rate of infiltration and their polarization state to therapeutic response. Furthermore, this underscores the potential of using macrophage polarization to stratify patients who are likely to fail standard immunotherapy. This data highlights the potential for many of the discussed cellular mediators as prognostic markers to stratify patients likely to fail standard immunotherapy. That said, the need to further solidify prognostic markers persists.

Our analysis categorized macrophage-targeted interventions into three primary focuses: M2 depletion, macrophage repolarization, and combination with existing immunotherapies. While M2 depletion physically reduces the immunosuppressive burden of anti-inflammatory macrophages, repolarization (e.g., via PI3Kγ) converts them from an immunosuppressive (e.g., C/EBP-B driven) to an immunostimulatory (e.g., NF-kB driven) M1 state without depleting the immune population. Current data and ongoing clinical trials support using these strategies alongside standard immunotherapies. However, the heavy reliance on preclinical models highlights the need for further translational research.

The macrophage-mediated resistance mechanisms described in this review do not operate in isolation but are embedded within a broader molecular resistance landscape that defines CRC biology. KRAS mutations, present in approximately 40% of CRC cases, are among the most consequential oncogenic drivers in this disease, promoting constitutive activation of the MAPK/ERK and PI3K/AKT pathways, suppressing anti-tumor immune signaling and contributing to the immunosuppressive TME that undermines the checkpoint blockade [94]. Importantly, oncogenic KRAS has been shown to functionally reprogram TAMs via CSF2 and lactate-mediated signaling, suggesting a direct mechanistic link between tumor cell–intrinsic oncogenic activity and macrophage polarization state. Alongside oncogene-driven resistance, adaptive resistance programs represent a parallel layer of therapeutic escape. YAP/TEAD signaling has emerged as a critical bypass mechanism in the setting of KRAS inhibition, with YAP1/TAZ activation compensating for suppressed KRAS effector output by restoring ERK-independent transcription of proliferative genes and activating the PI3K–AKT–mTOR axis [95]. Notably, the PI3K–AKT–mTOR axis identified as a downstream effector of TEAD-mediated resistance is the same pathway described in this review as a driver of M2 macrophage polarization, underscoring the convergence of oncogene-driven and immune resistance programs. Together, these observations reinforce that macrophage-targeted strategies in CRC must be designed with the tumor’s oncogenomic context in mind, and that durable responses will likely require co-targeting of both the immunosuppressive TME and the adaptive signaling networks that sustain tumor-cell survival.

Despite steps being taken to mitigate bias, limitations of the study design must be acknowledged. First, prioritizing recent studies in the rapidly evolving field of immunotherapy may have resulted in the omission of older foundational research. More importantly, centering the review exclusively on macrophage polarization introduces inherent confirmation bias. This focused target risks overestimating macrophages as therapeutic targets, potentially minimizing concurrent influence of other immune cells and mechanisms within the TME. With this, the strength of any clinical conclusions is currently limited by the paucity of completed randomized controlled trials (RCTs) specifically targeting these pathways. Much of the current data is derived from preclinical models or early-phase studies, and results from many active investigations are not yet publicly available for inclusion.

An additional limitation of these results is that while several prognostic and therapeutic findings are grounded in CRC-specific data (including SIRT-1-driven M2 polarization, CCL2/CCL4-mediated macrophage recruitment, IL-17A-mediated resistance, and CSF1R inhibition reprogramming TAMs in CRC models), some mechanistic conclusions are extrapolated from non-CRC tumor models. Specifically, PI3Kγ as a factor in immune suppression, T-cell exclusion driven by β-catenin, and CAF-mediated macrophage polarization toward a PD-L1 expressing phenotype have been implicated in non-CRC diseases. The biological plausibility of these mechanisms in CRC is supported by the shared immunosuppressive architecture of solid TMEs. Direct validation in CRC patient cohorts and specific preclinical models is an important next step in confirming their role in CRC as a focus of future translational work.

The strength of these findings is supported by the diversity of the analyzed papers, spanning preclinical studies to clinical investigation and trials. The theme of macrophage-mediated resistance evident across this breadth of study types suggests that the identified mechanisms provide robust biological relevance for clinical trials and prospective treatment approaches.

Future directions for this research are encouraged to translate the findings of preclinical data into actionable therapy. Several questions remain unanswered given our investigation, including (1) which specific subsets of CRC patients, particularly across different consensus molecular subtype (CMS) classifications, would benefit most from macrophage polarization therapy; (2) the standardized clinical prognostic value of the various markers discussed; (3) the mechanism by which the various pathways discussed operate within ICB therapy and contribute to states where TAM polarization are implicated. Future research should dive further into the TAM markers discussed and use spatial and transcriptomic signatures to identify likely responders to treatment. To guide patient selection, robust predictive biomarkers must be validated beyond traditional MSI/MSS testing. Future clinical trials must determine which distinct TMEs benefit most from TAM-repolarizing therapies. This includes addressing the gap as to the optimal timing and sequencing that such therapies would coincide with ICB therapy.

## 5. Conclusions

Macrophages significantly influence the TME in CRC by utilizing signaling cascades and cytokine networks to maintain immunotherapy resistance. The balance between pro- and anti-inflammatory macrophage polarization is a critical determinant of patient prognosis. Current trials focus on using blockades and agonists to shift the macrophages of the TME toward a pro-inflammatory state. These insights provide a foundation for combination therapies, pairing macrophage-targeted agents with standard immunotherapy, and identifying new biomarkers to advance personalized treatment for CRC patients.

## Figures and Tables

**Table 1:** Colorectal cancer microsatellite stability and mismatch repair status in immunotherapy.

	COLORECTAL CANCER GENETIC CLASSIFICATION	
Features	Microsatellite Stable/Mismatch Repair Proficient (MSS/MMRp)	Microsatellite Instability High/Mismatch Repair Deficient (MSI-H/MMRd)
TAM phenotype	High infiltration of M2 (anti-inflammatory) macrophages.	Higher infiltration of M1 (pro-inflammatory) macrophages.
Immune Checkpoint Protein Expression	Low PD-L1 expression.	Increased PD-L1, CTLA-4, LAG-3, and TIM-3 expression.
Other Key Characteristics	Low tumor mutational burden, chromosomal instability, KRAS enriched mutations, and metabolic dysregulation. T-cell exhausted TME. Patients respond better to standard chemotherapy. Prevalent in 80–85% CRC cases.	High tumor mutational burden. Responsive to anti-PD1/PDL1 immunotherapy. Prevalent in 10–15% of CRC cases.
Other Immune Cell Infiltrates	Marked absence of immune cell infiltration associated with “immune-cold” phenotype [19].	Higher infiltration of tumor-infiltrating lymphocytes, and resting NK cell association with “immune hot” phenotype [16].

**Table 2:** Colorectal cancer prognostic markers relevant to tumor-associated macrophage polarization.

Marker	Category	Prognosis	CRC Specificity	Study Type	Key Finding
M1:M2 ratio [47]	M1/M2 polarization	Favorable	CRC-direct	Clinical	Multiomics analysis of 2081 CRC tumors. Higher M1:M2 ratio in TME associated with improved patient survival across multiple observational studies.
Hepcidin/CD206 [48]	M1/M2 polarization	Unfavorable	CRC-direct	Clinical: Paired tissue from tumor and adjacent normally from sporadic CRC (*n* = 26). Findings validated with RNA expression analysis of 286 CRC samples.	Hepcidin protein expression was upregulated in tumor versus normal, and hepcidin RNA expression correlated with M2 macrophage infiltration markers including CD206 and IL-10. Increased tumor hepcidin expression was associated with reduced overall (OS) and disease-free survival (DFS).
SPP1 (Osteopontin) [49]	M1/M2 polarization	Unfavorable	Extrapolated	Bioinformatics analysis of gastric cancer (GC) GEO datasets GSE26943 (*n* = 205 GC, 58 normal), GSE13811 (*n* = 38 GC, 12 normal, GSE118916 *n* = 15 GC, 15 normal)	Differential expression analysis identified SPP1 upregulation in tumor vs. normal, an M2-like marker; SPP1 expression was significantly correlated with PDL1 expression, suggesting a potential predictive role for gastric cancer response to immunotherapy.
SIRT1 [31]	Intracellular regulator	Unfavorable	CRC-direct	Clinical: CRC tissue and peripheral blood. Preclinical: Co-culture model with CD8+ T cells, monocytes, and organoids.	Immunofluorescence analysis of tumor tissue demonstrated high SIRT1 expression on colon cancer cells, influencing TAM recruitment and CD8+ T-cell expression. High SIRT1 expression drives M2 polarization and inhibits CD8+ infiltration via CXCR4/CXCL12 axis and is associated with immunosuppressive cytokine activity (IL-10, TGF-β); RR = 2.459 (95% CI 1.315–4.469), *p* = 0.003
CSF1R [50]	Surface receptor	Unfavorable	CRC-direct	Preclinical: Murine model. Clinical: Survival data from open databases, inc. GSE39582, GSE17538 datasets, *n* = 232 patients.	CSF1R inhibition (PXB17) reprogrammed M2-like TAMs to M1 phenotype and enhanced the therapeutic benefit of anti-PD1 antibody in a murine model of both MSS and MSI-H CRC. CRC patient cohort survival analysis identified that increased tumor CSF1R expression is associated with poor prognosis (low vs. high expression, OS: HR 1.80 (95% CI 1.08–3.03), *p* = 0.023; RFS: HR 3.55 (95% CI 1.39–9.04), *p* = 0.0046)
EGFR (on TAMs) [51]	Surface receptor	Unfavorable	CRC-direct	Preclinical: Colon cancer-conditioned media treatment of bone marrow derived macrophages and tumor xenograft mouse model.	EGFR signaling promotes polarization of macrophages towards M2 phenotype, and inhibiting EGFR signaling pathways was found to reduce M2 polarization and macrophage-induced xenograft tumor growth in a colon cancer mouse model.
miRNA-146b, miNRA-155, miRNA-22 [52]	miRNA	Favorable	CRC-direct	Bioinformatics: Tumor bulk RNAseq from TCGA-CRC cohort, *n* = 406 colon (COAD), and *n* = 143 (READ) patients.	miRNA expression correlated with M1 macrophage infiltration of the TME in CRC and increased tumor PDL1 expression. Pathway analysis also demonstrated modulation of TGF-β signaling, which may promote M1 polarization and enhance responsiveness to checkpoint blockade.
IL-8, TIE2, HGF (plasma) [53]	Soluble biomarker	Unfavorable	Extrapolated	Clinical: Plasma protein expression in esophageal cancer patients (*n* = 91) before and after ICI treatment	Baseline plasma level significantly associated with shorter survival and immunotherapy response prediction in esophageal cancer. IL8: OS: HR 1.920 (95% CI 1.438–2.565), *p* < 0.001; PFS: HR 1.761 (95% CI 1.354–2.292), *p* < 0.001. TIE2: OS: HR 3.212 (95% CI 1.259–8.198), *p* = 0.015; PFS: HR 2.326 (95% CI 1.051–5.150), *p* = 0.039. HGF: OS: HR 2.318 (95% CI 1.144–4.698), *p* = 0.022; PFS: HR 2.010 (95% CI 1.083–3.732), *p* = 0.028.
GM-CSF (tumor-derived) [54]	Soluble biomarker	Favorable	CRC-direct	Clinical: Primary sporadic CRC tissue (*n* = 1420 patients) Preclinical: Monocytes isolated from PBMCs and treated in cell-line co-culture models.	High GM-CSF tumor expression associated with improved survival in MMRp CRC (*p* < 0.0001); tumor cell-derived GM-CSF promotes M1 polarization (CD16 macrophage expression) with cytostatic tumor effects. Increased expression remained an independent favorable prognostic factor, especially in MMRp/low CD8+ CRC.
CD8+ T cells/CXCR3+ [55]	Immune cell infiltration	Favorable	CRC-direct	Clinical	High infiltration of CXCR3-expressing CD8+ T cells linked to favorable prognosis and high M1:M2 ratio in CRC; STAT3 ablation: increased CD8+ T-cell accumulation (*p* < 0.001)
Immunoscore (CD3+/CD8+) [56]	Immune cell infiltration	Favorable	CRC-direct	Clinical	Internationally validated measure of T-cell infiltration at tumor core and invasive margin; predicts recurrence, DFS, and OS in stage I–III colon cancer; combining with macrophage phenotyping proposed to enhance stratification; 5 yr OS: low IS 71% (95% CI 66–77%), intermediate 84% (81–87%), high 92% (89–95%); stage II high IS: best DFS/OS (*p* < 0.05); MSI: 45% high IS vs. MSS: 21% high IS.
CD47/SIRPα axis [57]	Innate immune checkpoint	Unfavorable	Mixed Sources	Clinical	CD47 overexpression on cancer cells inhibits macrophage phagocytosis; particularly expressed in advanced disease; no response to magrolimab+cetuximab in KRAS-mutant CRC (favorable in KRAS WT); CD47 vs. CD86: Spearman’s r = 0.356, *p* < 0.001; AUC = 0.862 (sensitivity 0.818, specificity 0.829); N stage and clinical stage: *p* < 0.001.
CD74 [58]	Innate immune checkpoint	Favorable	CRC-direct	Preclinical	Potential predictor of response to cadonilimab (PD-1/CTLA-4 bispecific antibody), supported by in vitro co-culture systems and murine models.

CRC specificity of data is indicated (CRC direct, mixed [CRC and extrapolated] model sources, or extrapolated data from relevant models); AUC: Area under the curve, CD: Cluster of differentiation, CI: Confidence interval, CSF1R: Colony-stimulating factor receptor-1, CTLA-4: Cytotoxic T-lymphocyte-associated protein, CXCL: C-X-C motif chemokine ligand, CXCR: C-X-C chemokine receptor, DFS: Disease-free survival, EGFR: Epidermal growth factor receptor, GM-CSF: Granulocyte-macrophage colony-stimulating factor, HGF: Hepatocyte growth factor, HR: Hazard ratio, IL: Interleukin, iNOS: Inducible nitric oxide synthase, IS: Immunoscore, MAF: Musculoaponeurotic fibrosarcoma oncogene homolog, MRC1:Mannose receptor C-type 1, OR: Odds ratio, OS: Overall survival, PD-1: Programmed cell death protein 1, PDL1: Programmed death-ligand 1, PFS: Progression-free survival, RFS: Recurrence-free survival, RR: Relative risk, SIRPα: Signal regulatory protein alpha, SIRT1: Sirtuin 1, SPP1: Secreted phosphoprotein 1, STAT: Signal transducer and activator of transcription, TIE2: Tyrosine kinase with immunoglobulin and EGF homology domains 2, TME: Tumor microenvironment, TCGA: The Cancer Genome Atlas. ICI: Immune checkpoint inhibitors.

**Table 3:** Summary of clinical trial targets for macrophage polarization in colorectal cancer *.

**Therapy Class**	**Target/Pathway**	**TAM Effect**	**Combination(s)**	**Clinical Phase**	**Trials Involved**
Co-stimulatory agonists	CD137 (4-1BB)	Enhances co-stimulatory activity and dynamic immune monitoring	Anti-CTLA-4, Anti-PD-1/Anti-PDL-1	Phase 1/2	NCT04903873, NCT04121676, NCT03792724, NCT03290937
Cytokine-Based Therapy	IFNγ/IL12/IL15	Reshapes M1 polarization; induces Th1 response and cytotoxic activation	Anti-PD1/PDL1, antimetabolite chemotherapy, HDAC inhibition, PD-L1 + TGF-β inhibition	Phase 1/2	NCT03030378, NCT04708470, NCT04491955, NCT04613492, NCT04471987, NCT05352750, NCT04261439, NCT05619172, NCT04250155, NCT04616196, NCT03228667
TGF-β Targeting	TGF-β, CD39, TIGIT	Modulation of TGF-beta signaling and immune activation	Anti-PD-1/PDL-1, PD-L1 + TGF-β inhibition, recombinant IL-2, chemotherapy	Phase 1/2	NCT03436563, NCT03834662, NCT05537051, NCT05028556, NCT05381935, NCT04862767
Metabolic Modulators	Arginase inhibitors, CCR2/CCR5	Reverse immunosuppression by MDSCs and repolarize macrophages	Anti-PD-1, anti-CTLA4, chemotherapy	Phase 1/2	NCT02903914, NCT03184870, NCT03274804, NCT04721301
Macrophage Depletion	CSF-1R	Depletion of immunosuppressive tumor-associated macrophages (TAMs)	Anti-PD-1, anti-CD-40, mitotic inhibition chemotherapy	Phase 1/2	NCT02829723, NCT02452424, NCT02713529, NCT02880371
Phagocytosis Promoters	Anti-CD47/SIRPα antibodies	Blocks phagocytosis preventing signal on tumor cells to promote macrophage phagocytosis	EGFR inhibition, anti-PD-1, Nivolumab	Phase 1/2	NCT02953782, NCT03990233
TLR Agonists	TLR3, TLR7	Intratumoral immune activation and immunogenic cell death	Anti-CTLA4, Anti-PD-1/PDL-1, PD-1/CD47 antibodies, chemotherapy	Phase 1/2	NCT04588324, NCT04799054
Intracellular Signaling	TREM2, CD40 agonism	Myeloid cell modulation and reprogramming to overcome tumor-induced immunosuppression	Anti-PD-1/PDL-1, Anti-VEGF, chemotherapy, radiotherapy	Phase 1/preclinical	NCT04691375, NCT00607048, NCT02376699, NCT02665416, NCT05165433, NCT03555149, NCT04130854, NCT02600949, NCT03428126
Scavenger Receptor Targeting Cytokine-Based Therapy	MARCO/IFNγ/IL12	Reprograms TAMs toward pro-inflammatory chemokines; increases immune infiltration; Reshapes M1 polarization; induces Th1 response and cytotoxic activation	Anti-CTLA-4, Anti-PD-1, HAIP chemotherapy, HDAC inhibitors	Preclinical phase 1/2	Preclinical
Scavenger Receptor Targeting Intracellular Signaling	STING/TREM2/MARCO	GB2-induced M1 repolarization; enhances phagocytosis and T-cell infiltration	Anti-PD-1, Anti-CTLA-4	Preclinical phase 1	Preclinical

* See Supplemental Table S1 for details of selected list of clinical trials corresponding to Table 3 treatment categories.

## Data Availability

No new data were created or analyzed in this study.

## Supplementary Materials

The following supporting information can be downloaded at: https://www.mdpi.com/article/10.3390/cancers18132049/s1. Table S1: Clinical trials relevant to macrophage polarization therapy for colorectal cancer. Table S2: Risk of bias and relevance assessments; File S1: PRISMA 2020 Checklist.
