# Supplementary material for: Macrophage Polarization as a Target for Colorectal Cancer Treatment Optimization: A Systematic Review
Source: Cancers (Basel). 2026 Jun 24;18(13):2049. doi: 10.3390/cancers18132049 (PMC13360607; doi:10.3390/cancers18132049)
Supplement: Supplementary file 1 [file cancers-18-02049-s001.zip › File S1.pdf]

PRISMA 2020 Checklist

Macrophage Polarization as a Target for Colorectal Cancer Treatment Optimization: A Systematic Review

Seraphine et al., Cancers 2026 | PROSPERO ID: CRD420251244320

Section and Topic	#	Checklist Item	Location in Manuscript
**TITLE**			
Title	1	Identify the report as a systematic review.	**Title**
**ABSTRACT**			
Abstract	2	See the PRISMA 2020 for Abstracts checklist.	**Abstract (Background, Methods, Results, Conclusion)**
**INTRODUCTION**			
Rationale	3	Describe the rationale for the review in the context of existing knowledge.	**Introduction (Section 1)**<br>Describes CRC burden, limitations of ICB for MMRp/MSS tumors, role of TAMs in the TME, and gaps in existing synthesis of the literature.
Objectives	4	Provide an explicit statement of the objective(s) or question(s) the review addresses.	**Introduction (Section 1, final paragraph)**<br>The objective of this systematic review was to analyze the literature regarding the role of TAMs in the CRC response to immunotherapy.
**METHODS**			
Eligibility criteria	5	Specify the inclusion and exclusion criteria for the review and how studies were grouped for the syntheses.	**Methods, Sections 2.2–2.3**<br>Inclusion: original research/reviews on macrophages in immunotherapy response in cancer, microsatellite stability discussion. Exclusion: studies not focused on TAM and immunotherapy response, chemotherapy-only studies, editorials, commentaries, protocols, conference abstracts, letters.
Information sources	6	Specify all databases, registers, websites, organizations, reference lists and other sources searched or consulted to identify studies. Specify the date when each source was last searched or consulted.	**Methods, Section 2.1**<br>PubMed, EMBASE, and ScienceDirect searched from inception to December 2025. Additional articles identified from review articles and reference lists of selected studies.
Search strategy	7	Present the full search strategies for all databases, registers and websites, including any filters and limits used.	**Methods, Section 2.1**<br>Search terms: "macrophages" AND "immunotherapy" OR "immune checkpoint expression" AND "cancer" OR "microsatellite stability" OR "microsatellite instability". This string was used for PubMed, Science Direct and EMBASE.
Selection process	8	Specify the methods used to decide whether a study met the inclusion criteria of the review, including how many reviewers screened each record and each report retrieved, whether they worked independently, and if applicable, details of automation tools used in the process.	**Methods, Section 2.1**<br>Title/abstract screening performed independently by CS, TT, SD. Full-text review and inclusion by CS, TT, BM independently, with consensus reached through discussion. Endnote 21 (Clarivate) used to manage screening, duplicates, and reference management.
Data collection process	9	Specify the methods used to collect data from reports, including how many reviewers collected data from each report, whether they worked independently, any processes for obtaining or confirming data from	**Methods, Section 2.4**<br>Data extracted independently by CS, TT, BM using a predefined standardized form covering study design, patient/subject population, key outcomes, and

Section and Topic	#	Checklist Item	Location in Manuscript
		study investigators, and if applicable, details of automation tools used in the process.	relevance to cancer treatment. Endnote 21 used for screening process management.
Data items	10a	List and define all outcomes for which data were sought. Specify whether all results that were compatible with each outcome domain in each study were sought (e.g. for all measures, time points, analyses), and if not, the methods used to decide which results to collect.	**Introduction (Section 1); Results (Section 3, opening)** Three outcome domains defined: (1) macrophage-mediated mechanisms of resistance to ICB; (2) macrophage polarization status and biomarkers as prognostic indicators; (3) macrophage-targeted therapeutic strategies in clinical trials. Formal outcome-level specification not explicitly pre-stated in Methods.
Data items	10b	List and define all other variables for which data were sought (e.g. participant and intervention characteristics, funding sources). Describe any assumptions made about any missing or unclear information.	**Methods, Section 2.4** Standardized form recorded: study design, patient/subject population, relevant results, key outcomes, and relevance to cancer treatment. Cancer type and model system recorded during data extraction (noted in Results). Formal assumptions about missing data not described.
Study risk of bias assessment	11	Specify the methods used to assess risk of bias in the included studies, including details of the tool(s) used, how many reviewers assessed each study and whether they worked independently, and if applicable, details of automation tools used in the process.	**Methods, Section 2.5** Four tools applied: (1) SYRCLE Risk of Bias Tool for animal studies (n=19); (2) Structured four-domain framework for bioinformatics/database analyses (n=6); (3) Cochrane Risk of Bias Tool for RCTs (n=9); (4) Newcastle-Ottawa Scale for cohort/observational studies (n=19). Two reviewers independently assessed all studies; discrepancies resolved by consensus.
Effect measures	12	Specify for each outcome the effect measure(s) (e.g. risk ratio, mean difference) used in the synthesis or presentation of results.	**Not applicable — narrative synthesis only; no meta-analysis performed.** The review employs qualitative thematic synthesis. No statistical pooling of effect estimates was conducted.
Synthesis methods	13a	Describe the processes used to decide which studies were eligible for each synthesis (e.g. tabulating the study intervention characteristics and comparing against the planned groups for each synthesis).	**Methods, Sections 2.1–2.3; Results, Section 3 (opening)** Included studies were grouped into three thematic syntheses (mechanisms of resistance, prognostic markers, therapeutic strategies) based on their primary focus as identified during full-text screening and data extraction.
Synthesis methods	13b	Describe any methods required to prepare the data for presentation or synthesis, such as handling of missing summary statistics, or data conversions.	**Methods, Section 2.4** Data extracted to a predefined standardized form. No specific methods for handling missing summary statistics or data conversions are described beyond use of the extraction form.
Synthesis methods	13c	Describe any methods used to tabulate or visually display results of individual studies and syntheses.	**Results, Section 3; Figure 1; Table 1** PRISMA flow diagram (Figure 1) displays study selection. Table 1 provides a breakdown of MMR/MSI status. Table 2 discussed prognostic markers with effect estimates. Table 3 summarizes clinical trial targets. Figures 2 and 3 illustrate mechanistic pathways. Results organized narratively by thematic section.
Synthesis methods	13d	Describe any methods used to synthesize results and provide a rationale for the choice(s). If meta-analysis was performed, describe the model(s), method(s) to identify the presence and extent of statistical heterogeneity, and software package(s) used.	**Methods, Section 2 (implicit); Results, Section 3** Narrative/qualitative synthesis employed due to heterogeneity of study designs (mouse models, clinical samples, RCTs, bioinformatics analyses). Rationale for narrative approach not explicitly stated in the Methods section.
Synthesis methods	13e	Describe any methods used to explore possible causes of heterogeneity among study results (e.g. subgroup analysis, meta-regression).	**Not formally reported.** Study design heterogeneity acknowledged in Methods (Section 2.5) and Discussion (Section 4). No formal statistical heterogeneity exploration conducted.

Section and Topic	#	Checklist Item	Location in Manuscript
Synthesis methods	13f	Describe any sensitivity analyses conducted to assess robustness of the synthesized results.	**Not reported.**<br>No sensitivity analyses were conducted or described (consistent with narrative synthesis approach).
Reporting bias assessment	14	Describe any methods used to assess risk of bias due to missing results in a synthesis (arising from reporting biases).	**Not explicitly described in Methods.**<br>Reporting bias not formally assessed. Discussion (Section 4) acknowledges that results of many active trials are not yet publicly available for inclusion.
Certainty assessment	15	Describe any methods used to assess certainty (or confidence) in the body of evidence for an outcome.	**Not reported.**<br>No formal certainty assessment (e.g., GRADE) described or applied.
**RESULTS**			
Study selection	16a	Describe the results of the search and selection process, from the number of records identified in the search to the number of studies included in the review, ideally using a flow diagram.	**Results, Section 3 (opening paragraph); Figure 1**<br>4,115 papers identified; 3,687 screened after duplicate removal; 865 retrieved for full-text review; 65 included. PRISMA flow diagram presented as Figure 1.
Study selection	16b	Cite studies that might appear to meet the inclusion criteria, but which were excluded, and explain why they were excluded.	**Not reported.**<br>No table or list of excluded studies with reasons is provided in the manuscript or supplementary materials.
Study characteristics	17	Cite each included study and present its characteristics.	**Results, Sections 3.1–3.3; Supplementary Table 1**<br>Study types described by theme (e.g., Section 3.1: 6 mouse models, 10 patient/clinical studies, 2 preclinical, 2 computational). Individual studies cited with key characteristics described in context. Formal characteristics table not present in main text; Supplementary Table 1 covers clinical trials.
Risk of bias in studies	18	Present assessments of risk of bias for each included study.	**Results, Section 3.4; Supplementary Materials**<br>Per-study risk of bias results summarized narratively in Section 2.5 (specific studies named with ratings). Full risk of bias results reported in Supplementary Materials.
Results of individual studies	19	For all outcomes, present, for each study: (a) summary statistics for each group (where appropriate) and (b) an effect estimate and its precision (e.g. confidence/credible interval), ideally using structured tables or plots.	**Results, Sections 3.1–3.3**<br>Table 2 explicitly reports per-study effect estimates with confidence intervals and p-values for multiple markers; mechanistic and therapeutic-theme studies (Section 3.1 and 3.3) remain narrative without structure effect-estimate table
Results of syntheses	20a	For each synthesis, briefly summarize the characteristics and risk of bias among contributing studies.	**Results, Sections 3.1, 3.2, 3.3 (opening paragraphs)**<br>Each section opens with a count and breakdown of study design types contributing to that thematic synthesis (e.g., Section 3.1: 20 papers — 6 mouse models, 10 patient/clinical, 2 preclinical, 2 computational; Section 3.2: 4 preclinical + 7 clinical investigations).
Results of syntheses	20b	Present results of all statistical syntheses conducted. If meta-analysis was done, present for each the summary estimate and its precision (e.g. confidence/credible interval) and measures of statistical heterogeneity. If comparing groups, describe the direction of the effect.	**Results, Sections 3.1–3.3**<br>No statistical meta-analysis performed. Narrative synthesis results presented across Sections 3.1–3.3 by theme.
Results of syntheses	20c	Present results of all investigations of possible causes of heterogeneity among study results.	**Not formally reported.**<br>Study design heterogeneity acknowledged qualitatively in Discussion (Section 4) but not formally investigated.

Section and Topic	#	Checklist Item	Location in Manuscript
Results of syntheses	20d	Present results of all sensitivity analyses conducted to assess the robustness of the synthesized results.	**Not reported.** No sensitivity analyses conducted.
Reporting biases	21	Present assessments of risk of bias due to missing results (arising from reporting biases) for each synthesis assessed.	**Not explicitly reported.** Not formally assessed. Acknowledged in Discussion (Section 4) that many active trial results are not yet publicly available.
Certainty of evidence	22	Present assessments of certainty (or confidence) in the body of evidence for each outcome assessed.	**Not reported.** No formal certainty assessment applied to outcomes.
**DISCUSSION**			
Discussion	23a	Provide a general interpretation of the results in the context of other evidence.	**Discussion, Section 4** Synthesizes findings on immunosuppressive mechanisms, prognostic markers, and macrophage-targeted therapeutic strategies in the broader context of CRC immunotherapy research.
Discussion	23b	Discuss any limitations of the evidence included in the review.	**Discussion, Section 4** Acknowledges: paucity of completed RCTs targeting this pathway; heavy reliance on preclinical models; non-CRC extrapolations for several mechanistic conclusions (PI3Kγ, β-catenin, CAF-mediated polarization).
Discussion	23c	Discuss any limitations of the review processes used.	**Discussion, Section 4** Acknowledges: potential omission of older foundational research due to prioritization of recent studies; confirmation bias from exclusive focus on macrophage polarization; many active trials with results not yet publicly available.
Discussion	23d	Discuss implications of the results for practice, policy, and future research.	**Discussion, Section 4; Conclusions, Section 5** Recommends combination macrophage-targeted strategies with existing ICB; identifies need for further translational research and RCTs; highlights macrophage polarization markers for patient stratification.
**OTHER INFORMATION**			
Registration and protocol	24a	Provide registration information for the review, including register name and registration number, or state that the review was not registered.	**Abstract (Methods); Methods, Section 2.1; Reference 23** Prospectively registered on PROSPERO (ID: CRD420251244320).
Registration and protocol	24b	Indicate where the review protocol can be accessed, or state that a protocol was not prepared.	**Methods, Section 2.1; Reference 23** PROSPERO record accessible at: https://www.crd.york.ac.uk/PROSPERO/view/CRD420251244320
Registration and protocol	24c	Describe and explain any amendments to information provided at registration or in the protocol.	**Not reported.** No amendments to registration or protocol are described.
Support	25	Describe sources of financial or non-financial support for the review, and the role of the funders or sponsors in the review.	**Funding section; Acknowledgments** No external funding received. Non-financial support: Mary K. Oxley Foundation, Price/Bass Family, and John W. Price and Barbara Thruston Atwood Price Trust.
Competing interests	26	Declare any competing interests of review authors.	**Conflicts of Interest section** Susan Galandiuk receives a stipend from the American Society of Colon and Rectal Surgeons for editorial services at Diseases of the Colon & Rectum. No other disclosures reported.

Section and Topic	#	Checklist Item	Location in Manuscript
Availability of data, code and other materials	27	Report which of the following are publicly available and where they can be found: template data collection forms; data extracted from included studies; data used for all analyses; analytic code; any other materials used in the review.	**Data Availability Statement; Supplementary Materials** No new data were created or analyzed. PRISMA checklist in Supplementary Materials. Supplementary Table 1 (clinical trials) available at MDPI supplementary link.
