# Supplementary material for: Macrophage Polarization as a Target for Colorectal Cancer Treatment Optimization: A Systematic Review
Source: Cancers (Basel). 2026 Jun 24;18(13):2049. doi: 10.3390/cancers18132049 (PMC13360607; doi:10.3390/cancers18132049)
Supplement: Supplementary file 1 [file cancers-18-02049-s001.zip › Table S1.pdf]

Supplementary Table S1: Clinical trials relevant to macrophage polarization therapy for colorectal cancer.

Clinical Trial	Study Details (Status, Focus, Findings)
**CD137 Agonists**	
NCT04903873	**Status:** Ongoing<br>**Focus:** evaluating the safety, efficacy, and pharmacokinetics of EU101, an agonistic anti-CD137 (4-1BB) monoclonal antibody, in patients with advanced solid tumors<br>**Findings:** no results posted
NCT04121676	**Status:** Completed<br>**Focus:** evaluating the safety and efficacy of AGEN2373, a conditionally active CD137 agonist designed to mitigate liver toxicity, both as a monotherapy and in combination with botensilimab (an anti-CTLA-4 antibody) in patients with advanced solid tumors<br>**Findings:** results from the monotherapy arm demonstrated a favorable safety profile with no dose-limiting toxicities or hepatotoxicity and showed preliminary clinical activity, including durable partial responses in patients with refractory gynecologic and gastrointestinal cancers
NCT03792724	**Status:** Unknown<br>**Focus:** evaluating the safety and pharmacodynamic activity of intratumoral urelumab (anti-CD137 agonist) combined with systemic nivolumab (anti-PD-1) in patients with advanced solid tumors<br>**Findings:** published biomarker data indicate that the treatment consistently increased plasma levels of soluble CD137, indicating effective costimulatory activity
NCT03290937	**Status:** Completed<br>**Focus:** evaluating dose and side effects of irinotecan hydrochloride (topoisomerase I inhibitor) when given with utomilumab (Anti-CD137 agonist) and cetuximab (Anti-EGFR) in treating patients with metastatic colorectal cancer<br>**Findings:** No results posted
**Interferon-γ/interleukin 12**	
NCT03030378	**Status**: Active, not recruiting<br>**Focus**: evaluating safety and efficacy when combining pembrolizumab (a PD-1 inhibitor) with recombinant IL-12 (immunostimulatory cytokine) in patients with solid tumors<br>**Findings**: no results posted
NCT05286814	**Status**: Ongoing (recruiting)<br>**Focus**: evaluating the efficacy of PDS01ADC (tumor-targeted IL-12 immunocytokine) in combination with hepatic artery infusion pump (HAIP) therapy and systemic FUDR and Dexamethasone chemotherapy in patients with metastatic colorectal cancer or intrahepatic cholangiocarcinoma<br>**Findings**: No results posted
NCT04708470	**Status**: Active (recruiting)<br>**Focus**: evaluating the combination of bintrafusp-α (fusion protein targeting PD-L1 and TGF-β), PDS01ADC, and entinostat (histone deacetylase inhibitor) in patients with advanced HPV-associated malignancies and microsatellite stable colorectal/small bowel cancers<br>**Findings**: no results posted

NCT04491955	**Status**: Completed<br>**Focus**: evaluating the safety and efficacy of combining multiple immunotherapies— CV301 (poxviral vaccine targeting CEA and MUC1), N-803 (an IL-15 superagonist), bintrafusp-α, and M9241 (tumor-targeted IL-12 immunocytokine)—in patients with advanced microsatellite stable colorectal and small bowel cancers<br>**Findings**: combination therapy demonstrated a manageable safety profile and preliminary clinical activity, with some patients achieving disease reduction and promising 12-month overall survival rates compared to historical controls.
NCT04287868	**Status**: Active (not recruiting)<br>**Focus**: evaluating the combination of PDS0101 (HPV-16 therapeutic vaccine), M9241/NHS-IL12 (tumor-targeted IL-12 immunocytokine), and bintrafusp-α in patients with advanced HPV-associated malignancies<br>**Findings**: combination therapy showed a manageable safety profile and promising clinical activity, particularly in HPV-16 positive patients naïve to immune checkpoint blockade, with high objective response rates and overall survival
NCT02498912	**Status**: Active, not recruiting<br>**Focus**: evaluating the use of CAR T cells genetically engineered to secrete IL-12 and target the MUC16ecto antigen in patients with cyclophosphamide refractory MUC16ecto+ high-grade serous ovarian cancer<br>**Findings**: no results posted
NCT04613492	**Status**: Terminated<br>**Focus**: evaluating the safety and efficacy of MEDI9253 (recombinant oncolytic virus encoding IL-12) in combination with durvalumab (anti-PD-L1 antibody) in patients with advanced solid tumors<br>**Findings**: no results posted
NCT04471987	**Status**: Active (recruiting)<br>**Focus**: evaluating the safety and efficacy of IL12-L19L19 (antibody-cytokine fusion protein delivering interleukin-12 to the tumor neovasculature) in patients with advanced solid tumors following immune checkpoint blockade therapy<br>**Findings**: no results posted
NCT05352750	**Status**: Active (not recruiting)<br>**Focus**: evaluating SON-1010 (tumor-targeted recombinant human interleukin-12, linked to albumin-binding domain) as a monotherapy and in combination with trabectedin (DNA minor groove chemotherapy) in patients with advanced solid tumors<br>**Findings**: interim results from the monotherapy dose-escalation phase demonstrated a manageable safety profile with no dose-limiting toxicities, while showing preliminary clinical activity including stable disease in 48% of evaluable patients and one confirmed partial response.
NCT04261439	**Status**: Terminated<br>**Focus**: evaluating the safety and efficacy of NIZ985 (recombinant heterodimer of IL-15/IL-15 receptor alpha) alone and in combination with spartalizumab (anti-PD-1) or tislelizumab (anti-PD-1) in patients with advanced solid tumors and lymphoma<br>**Findings**: no results posted

NCT05619172	**Status**: Terminated (discontinued by sponsor)<br>**Focus**: evaluating the efficacy and safety of nanrilkefusp-α (SOT101, an IL-15 receptor beta/gamma agonist) in combination with cetuximab in patients with RAS wild-type colorectal cancer<br>**Findings:** interim data indicated that nanrilkefusp-α did not demonstrate sufficient efficacy as a monotherapy or in combination with other agents to warrant further development
NCT04250155	**Status**: Active (not recruiting)<br>**Focus**: evaluating the safety, pharmacokinetics, and activity of XmAb24306 (IL-15/IL-15 receptor alpha complex) alone and in combination with atezolizumab (anti-PD-L1) in patients with locally advanced or metastatic solid tumors<br>**Findings**: no results posted
NCT04616196	**Status**: Completed<br>**Focus**: evaluating the safety and efficacy of NKTR-255 (polymer-conjugated human IL-15 agonist designed to boost natural killer and CD8+ T-cell activity) in combination with cetuximab in patients with relapsed/refractory head and neck squamous cell carcinoma (HNSCC) or colorectal cancer (CRC)<br>**Findings**: combination was well-tolerated and biologically active, inducing the expansion of NK and CD8+ T cells; preliminary efficacy showed one confirmed partial response in a colorectal cancer patient and stable disease in two HNSCC patients.
NCT03228667	**Status:** Active (not recruiting)<br>**Focus:** evaluating combination therapy of nogapendekin alfa inbakicept (NAI, IL-15 superagonist fusion protein) with checkpoint inhibitor therapies in various cancers<br>**Findings:** no results posted
**TGF-B**	
NCT04432597	**Status**: Active (not recruiting)<br>**Focus**: evaluating PRGN-2009 (gorilla adenovirus-based HPV vaccine targeting E6 and E7) alone or in combination with bintrafusp-α (a bifunctional fusion protein targeting PD-L1 and TGF-β) in patients with HPV-associated cancers<br>**Findings**: no results posted
NCT03436563	**Status:** Completed<br>**Focus:** evaluating the safety and efficacy of bintrafusp-α/M7824 in patients with metastatic colorectal cancer or advanced MSI-H solid tumors<br>**Findings:** treatment demonstrated limited efficacy, potential adverse effects
NCT02723955	**Status:** Completed<br>**Focus:** evaluating the safety, pharmacology, and preliminary antitumor activity of feladilimab (GSK3359609, ICOS receptor agonist) alone and in combination with pembrolizumab (anti-PD-1) or various chemotherapy regimens in patients with advanced solid tumors<br>**Findings:** preliminary results showed the combination had a manageable safety profile and promising clinical activity, particularly in HNSCC; later phase trials in HNSCC were discontinued based on totality of data

NCT03834662	**Status**: Completed<br>**Focus**: evaluating the safety and efficacy of AVID200 (selective inhibitor of TGF-B1 & TGF-B3) alone and in combination with pembrolizumab (anti-PD-1) or durvalumab (anti-PD-L1) in patients with advanced solid tumors<br>**Findings**: no results posted
NCT05537051	**Status**: Unknown<br>**Focus**: evaluating the safety, tolerability, pharmacokinetics, and preliminary efficacy of PM1021 (anti-TIGIT) as a monotherapy and in combination with PM8001 (anti-PD-L1/TGF-B fusion protein) in patients with advanced solid tumors<br>**Findings**: no results posted
NCT05028556	**Status:** Completed<br>**Focus:** evaluating safety and efficacy of Y101D (PD-L1/ TGF-β Bispecific Antibody) in metastatic or locally advanced solid tumors<br>**Findings:** no results posted
NCT05381935	**Status**: Withdrawn<br>**Focus**: evaluating the safety, tolerability, and preliminary efficacy of ES014 (bispecific antibody targeting CD39 and TGF-B) in patients with locally advanced or metastatic solid tumors<br>**Findings**: no results posted
NCT04862767	**Status**: Completed<br>**Focus**: evaluating the safety, tolerance, and efficacy of TASO-001 (TGF-B2 targeting anti-sense oligonucleotide) in combination with recombinant aldesleukin (recombinant IL-2) in patients with advanced or metastatic solid tumors; goal of assessing dosing for phase 2 trial<br>**Findings**: no results posted
**Vitamin E**	
NCT04245865	**Status:** Active, not recruiting<br>**Focus:** evaluating the efficacy and safety of adding tocotrienol (vitamin E isomer) to bevacizumab (anti-VEGF) and standard chemotherapy (FOLFOX or capecitabine) in patients with metastatic colorectal cancer<br>**Findings:** no results posted
NCT02705300	**Status**: Completed<br>**Focus**: evaluating whether adding tocotrienol to FOLFOXIRI chemotherapy reduces treatment-related toxicities, specifically hospitalization and neuropathy, in patients with metastatic colorectal cancer<br>**Findings**: adding delta-tocotrienol to FOLFOXIRI did not significantly prolong the time to first hospitalization or death compared to placebo. It did not reduce the incidence of peripheral neuropathy or other grade 3-4 toxicities, though fewer patients in the tocotrienol group required oxaliplatin dose reductions.
**MEK Inhibition**	
NCT03428126	**Status**: Completed<br>**Focus**: evaluating the efficacy and safety of combining durvalumab (anti-PD-L1) and trametinib (MEK inhibitor) in patients with microsatellite-stable (MSS) metastatic colorectal cancer, with the goal of modulating the immune tumor microenvironment

	**Findings**: the study did not meet its efficacy criteria to proceed to the second stage; the overall response rate was 3.4% (1 out of 29 patients achieved a partial response), median progression-free survival was 3.2 months, and no significant changes in T-cell infiltration were observed in tumor biopsies
**Metabolic Inhibitors**	
NCT02903914	**Status:** Completed<br>**Focus:** evaluating the safety and efficacy of INCB001158 (small molecule arginase inhibitor) alone and in combination with pembrolizumab (anti-PD-1) in patients with advanced or metastatic solid tumors<br>**Findings:** treatment consistently increased plasma arginine levels and had a manageable safety profile but demonstrated limited antitumor activity with a lower objective response rate than controls
NCT03184870	**Status:** Completed<br>**Focus:** evaluating the safety and efficacy of BMS-813160 (dual CCR2/CCR5 antagonist) alone or in combination with chemotherapy (gemcitabine/nab-paclitaxel or FOLFIRI) or nivolumab (anti-PD-1) in patients with advanced metastatic colorectal and pancreatic cancers<br>**Findings:** results showed that therapy was generally well tolerated; results were initially promising in pancreatic cancer, but lacked efficacy in MMS colorectal cancer
NCT03274804	**Status** Completed (Phase 1)<br>**Focus** Investigating whether combining Maraviroc (CCR5 antagonist) with pembrolizumab can modulate tumor-associated macrophages to overcome immune resistance in refractory microsatellite-stable (MSS) metastatic colorectal cancer<br>**Findings** combination therapy demonstrated a good safety profile but limited clinical activity, achieving an Objective Response Rate (ORR) of 5.3% and a median Overall Survival of 9.8 months; translational data suggested successful modulation of the tumor microenvironment despite the modest clinical response
NCT04721301	**Status:** Completed<br>**Focus:** evaluated Maraviroc combined with Nivolumab (anti-PD-1) and Ipilimumab (anti-CTLA-4)<br>**Findings:** no results posted
**CSF1R Inhibitors**	
NCT02829723	**Status:** Terminated<br>**Focus:** Phase 1/2 study evaluating the safety and efficacy of BLZ945 (CSF-1R inhibitor) alone or in combination with Spartalizumab (PDR001, an anti-PD-1 antibody) in patients with advanced solid tumors (specifically targeting tumor associated macrophages)<br>**Findings:** Although the treatment successfully modulated the tumor microenvironment, this translated to limited clinical efficacy with the combination showing insufficient antitumor activity to warrant further development in the tested settings
NCT02452424	**Status:** Terminated (Phase 1/2a)<br>**Focus:** Evaluating the combination of PLX3397 (CSF-1R inhibitor) and pembrolizumab (PD-1 inhibitor) to deplete tumor-associated macrophages (TAMs) and overcome immune suppression in patients with advanced melanoma and other solid tumors<br>**Findings:** terminated early due to insufficient evidence of clinical efficacy; depleting macrophages with PLX3397 did not sufficiently enhance the antitumor activity of pembrolizumab in this setting to warrant further development
NCT01494688	**Status:** Completed (Phase 1)<br>**Focus:** Evaluating RO5509554 (anti-CSF-1R monoclonal antibody) alone or in combination with Paclitaxel in patients with advanced solid tumors, specifically targeting the depletion of tumor-associated macrophages (TAMs)

	**Findings:** no results posted
NCT02760797	**Status:** Completed (Phase 1b)<br>**Focus:** Evaluating the combination of Emactuzumab (anti-CSF-1R monoclonal antibody) and Selicrelumab (RO7009789, a CD40 agonist) in patients with advanced solid tumors, hypothesizing that concurrent macrophage depletion (via CSF-1R inhibition) and immune activation (via CD40 agonism) would enhance antitumor immune responses.<br>**Findings:** no results posted
NCT02713529	**Status:** Completed (Phase 1b/2)<br>**Focus:** Evaluating the safety and efficacy of AMG 820 (anti-CSF-1R antibody) in combination with pembrolizumab for patients with advanced solid tumors (specifically non-small cell lung cancer, colorectal cancer, and pancreatic cancer), aiming to reduce immunosuppressive tumor-associated macrophages.<br>**Findings:** combination demonstrated an acceptable safety profile but failed to achieve meaningful clinical efficacy, with no cohorts meeting the predefined thresholds to warrant further development
NCT02880371	**Status:** Completed (Phase 1b/2)<br>**Focus:** Evaluating the safety and efficacy of ARRY-382 (selective CSF-1R inhibitor) in combination with pembrolizumab (PD-1 inhibitor) for patients with advanced solid tumors, specifically targeting pancreatic ductal adenocarcinoma, platinum-resistant ovarian cancer, and PD-1/L1-refractory tumors<br>**Findings:** the combination was well tolerated, but limited clinical benefit was observed
**Anti-CD47/SIRPα antibodies**	
NCT02953782	**Status:** Completed (Phase 1b/2)<br>**Focus:** evaluated the combination of Magrolimab (Hu5F9-G4, anti-CD47) and Cetuximab (anti-EGFR)<br>**Findings:** The combination was generally safe and exhibited potential for patients with extensive initial treatment
NCT03990233	**Status:** Completed<br>**Focus:** This trial evaluated BI 765063 (SIRPα antagonist) both as a monotherapy and in combination with BI 754091 (PD-1 checkpoint inhibitor)<br>**Findings:** no results posted
**TLR 3 Agonist**	
NCT04508140	**Status:** Terminated<br>**Focus:** Evaluating the safety and efficacy of intratumoral BO-112 (synthetic dsRNA/TLR3 agonist) in combination with pembrolizumab (anti-PD-1) to reverse resistance in patients with advanced colorectal (CRC) or gastric/GEJ cancer with liver metastases<br>**Findings:** no results posted, terminated due to low recruitment rate
**TLR7 Agonists**	
NCT04588324	**Status:** Unknown (Phase 1/2)<br>**Focus:** Evaluating SHR2150 (a TLR7 agonist) combined with first-line cytotoxic agents and either a PD-1 antibody or CD47 antibody in patients with unresectable or metastatic solid tumors<br>**Findings:** no results posted; last updated as recruiting (2020)

NCT04799054	**Status:** Terminated<br>**Focus:** Evaluating the safety, tolerability, and preliminary efficacy of TransCon TLR7/8 Agonist (a sustained-release prodrug of resiquimod administered intratumorally) as a monotherapy or in combination with pembrolizumab (PD-1 inhibitor) in patients with locally advanced or metastatic solid tumors<br>**Findings:** though appearing well-tolerated with potential efficacy, sponsor terminated to prioritize other efforts
NCT04278144	**Status:** Terminated (Phase 1/2).<br>**Focus:** evaluating safety and efficacy of BDC-1001 (HER2-targeting Immune-Stimulating Antibody Conjugate, ISAC) as a single agent and in combination with Nivolumab (anti-PD-1) in patients with advanced HER2-expressing solid tumors (including breast, colorectal, and others)<br>**Findings:** despite promising activity and safety, sponsor terminated to focus efforts elsewhere
**TREM2 Inhibitors**	
NCT04691375	**Status:** Terminated<br>**Focus:** Evaluating the safety, tolerability, and pharmacodynamics of PY314 (anti-TREM2 monoclonal antibody) as a single agent and in combination with pembrolizumab (anti-PD-1) in patients with advanced solid tumors<br>**Findings:** no results posted; terminated due to sponsor business decision
**Clever 1 Inhibitors**	
NCT03733990	**Status:** Completed<br>**Focus:** Evaluating the safety, tolerability, and preliminary efficacy of Bexmarilimab (anti-CLEVER-1 antibody targeting macrophages) as a monotherapy in patients with advanced refractory solid tumors<br>**Findings:** therapy well-tolerated with no dose-limiting toxicities observed. Showed promising results in immune activation in selected patients, particularly in patients with low immune baselines.
**Complement Inhibitors**	
NCT03665129	**Status:** Terminated<br>**Focus:** Evaluating the safety, tolerability, and antitumor activity of IPH5401 (anti-C5aR antibody) in combination with Durvalumab (anti-PD-L1) in patients with selected advanced solid tumors, including non-small cell lung cancer (NSCLC), hepatocellular carcinoma (HCC), and other solid malignancies.<br>**Findings:** no results posted
**Macrophage Cell Therapy**	
NCT04660929	**Status:** Active, not recruiting (Phase 1)<br>**Focus:** evaluating CT-0508 (anti-HER2 CAR-Macrophage) alone or with Pembrolizumab for recurrent HER2-overexpressing solid tumors.<br>**Findings:** no results posted
**Agonist anti-CD40 antibodies**	
NCT00607048	**Status:** Completed (Phase 1)<br>**Focus:** evaluating the safety and efficacy of CP-870,893 (a CD40 agonist antibody) in combination with Paclitaxel and Carboplatin in patients with advanced solid tumors, particularly focusing on metastatic melanoma<br>**Findings:** Demonstrated safety and efficacy for patients with advanced solid tumors

NCT02376699	**Status:** Terminated<br>**Focus:** Evaluating the safety and efficacy of SEA-CD40 (non-fucosylated CD40 agonist) alone or in combination with Pembrolizumab and chemotherapy (Gemcitabine + Nab-paclitaxel) in patients with advanced solid tumors, specifically targeting metastatic Pancreatic Ductal Adenocarcinoma (PDAC)<br>**Findings:** terminated due to portfolio prioritization, no results posted
NCT02665416	**Status:** Completed (Phase 1)<br>**Focus:** A two-part study evaluating the safety, pharmacokinetics, and therapeutic activity of Selicrelumab (RO7009789, a CD40 agonist) in combination with Vanucizumab (a bispecific anti-Ang2/VEGF antibody) or Bevacizumab (anti-VEGF) in patients with metastatic solid tumors.<br>**Findings:** The combination of subcutaneous Selicrelumab and Vanucizumab demonstrated a favorable safety profile and early signs of clinical activity in patients with advanced solid tumors; however, the development of Vanucizumab was discontinued, leading to a switch to Bevacizumab for the expansion cohorts
NCT05165433	**Status:** Completed (Phase 1a/1b)<br>**Focus:** evaluating NG-350A (tumor-selective adenoviral vector expressing an anti-CD40 agonist antibody) alone (intravenous or intratumoral) and in combination with Pembrolizumab (PD-1 inhibitor) in patients with metastatic or advanced epithelial tumors<br>**Findings:** Indicated that NG-350A is well-tolerated with no dose-limiting toxicities or off-target viral effects observed; it demonstrated successful tumor-selective delivery, viral replication, and transgene expression (anti-CD40) in tumor biopsies, along with sustained increases in inflammatory cytokines, particularly with intravenous dosing
NCT03555149	**Status:** Terminated<br>**Focus**: evaluating the safety and efficacy of multiple immunotherapy-based combinations in patients with metastatic colorectal cancer (mCRC), primarily focusing on refractory microsatellite-stable (MSS) disease to identify signals of clinical activity<br>**Findings:** limited efficacy shown; terminated due to recruitment and resource issues
NCT04130854	**Status:** Active (not recruiting)<br>**Focus:** evaluated the safety and efficacy of adding sotigalimab (APX005M, a CD40 agonist) to neoadjuvant Short-Course Radiation Therapy (SCRT) followed by mFOLFOX chemotherapy in patients with locally advanced rectal cancer<br>Findings: no results posted
NCT02600949	**Status:** Active (not recruiting – Phase 1)<br>**Focus:** evaluating the safety and feasibility of a personalized neoantigen peptide vaccine peptide-based vaccine in combination with imiquiomod, pembrolizumab, or sotigalimab (APX005M) in patients with advanced pancreatic and MMS metastatic colorectal cancer<br>**Findings:** no results posted
