# Supplementary material for: Macrophage Polarization as a Target for Colorectal Cancer Treatment Optimization: A Systematic Review
Source: Cancers (Basel). 2026 Jun 24;18(13):2049. doi: 10.3390/cancers18132049 (PMC13360607; doi:10.3390/cancers18132049)
Supplement: Supplementary file 1 [file cancers-18-02049-s001.zip › Table S2.pdf]

Supplementary Table S2. Risk of bias and relevance assessments.

a. Murine Studies (SYRCLE Risk of Bias Tool)

**Study**	**Reviewer Consensus**
Chen, 2020	High risk
Joshi, 2020	High risk
Fang, 2022	Moderate risk
Kaneda, 2016 †	Low risk
Fontenot, 2003	Moderate risk
Denk, 2025	High risk
Loke, 2003	High risk
Peng, 2016	Moderate risk
Guo, 2023	Low risk
Yue, 2015 †	High risk
Acha-Sagredo, 2025	Moderate risk
Deng, 2025	Low risk
Georgoudaki, 2016	Moderate risk
Katzenelenbogen, 2020	Moderate risk
Zhang, 2025	Moderate risk
Tadayon, 2021	Low risk
Zhang, 2020	Low risk
Zhang, 2016	
Zhao, 2021	Moderate risk
Yuan, 2022	Low risk
Wang, 2024	Moderate risk

Color key: Low risk Moderate Risk High Risk

† Inter-reviewer discrepancy resolved by consensus discussion. Ratings assigned as Low, Moderate, or High risk of bias assessed across ten SYRCLE domains: (1) randomization of animal allocation, (2) blinding of personnel, (3) blinding of outcome assessors, (4) completeness of outcome data, (5) selective reporting, (6) other bias, (7) animal selection bias, (8) animal handling and care, (9) environmental control, and (10) statistical analysis.

b. Cohort and Observational Studies (Newcastle-Ottawa Risk Scale)

Study	Reviewer Consensus
Gao, 2024	Moderate risk
Tian, 2024	Moderate risk
Mezheyeuski, 2023	Moderate risk
Spranger, 2015	Moderate risk
Sica, 2000	Low risk
Wang, 2019	Low risk
De la Fuente Lopez, 2018	Low risk
Chalmin, 2010 †	High risk
Edin, 2012	Moderate risk
Gok Yavuz, 2019	Low risk
Llosa, 2019	Low risk
Di Grazia, 2022	Low risk
Pagès, 2018	Low risk
Lv, 2024 †	Low risk
Chen, 2014	Low risk
Nebiker, 2014	Moderate risk
Min, 2021 †	Low risk
Cabrales, 2019	High risk
Maeda, 2019	Moderate risk
Zhu, 2021	Moderate risk

Color key: Low risk Moderate Risk High Risk

† Inter-reviewer discrepancy resolved by consensus discussion. Aggregate Newcastle-Ottawa Scale (NOS) point scores converted to risk categories, assessed across three domains: selection of study groups, comparability of cohorts, and adequacy of outcome ascertainment.

c. Methodological Quality and Relevance Assessment of Bioinformatics and Database Studies

Study	Reviewer Consensus
Peng, 2025	High
Bao, 2024	High
Ugai, 2022	High

Study	Reviewer Consensus
Zhong, 2022	High
Xie, 2025	High
Bartlett, 2021	High
Wang, 2021	High

Color key: High relevance Moderate relevance Low relevance

Ratings reflect methodological quality and relevance (High, Low, or Unclear) rather than traditional risk of bias, assessed across four domains: (1) data source quality, (2) analytical transparency, (3) external validation, and (4) outcome relevance. No inter-reviewer discrepancies were identified.

d. Randomized Controlled Trials (Cochrane Risk of Bias Tool)

Study	Reviewer Consensus
Van Cutsem, 2009	Low risk
Eng, 2025	Low risk
Morris, 2022	Low risk
Raunkilde, 2023	Low risk
Johnson, B, 2022	Low risk
Naing, 2024	Low risk
Le, 2026	Low risk
Haag, 2022	Low risk
Li, 2022	Low risk
Montagne, 2025	Low risk
Razak, 2020	Low risk
Johnson, M, 2022	Low risk

Color key: Low risk Moderate Risk High Risk

Ratings assigned as Low, Moderate, or High risk of bias across Cochrane RoB domains: randomization sequence generation, allocation concealment, blinding of participants and personnel, blinding of outcome assessors, completeness of outcome data, selective outcome reporting, and other bias. No inter-reviewer discrepancies were identified.
